# Photochemical Generation and Characterization of *C*-Aminophenyl-Nitrilimines: Insights on Their Bond-Shift Isomers by Matrix-Isolation IR Spectroscopy and Density Functional Theory Calculations

**DOI:** 10.3390/molecules29153497

**Published:** 2024-07-25

**Authors:** A. J. Lopes Jesus, Cláudio M. Nunes, Gil A. Ferreira, Kiarash Keyvan, R. Fausto

**Affiliations:** 1 University of Coimbra, CQC-IMS, Faculty of Pharmacy, 3004-295 Coimbra, Portugal; 2 University of Coimbra, CQC-IMS, Department of Chemistry, 3004-535 Coimbra, Portugal; cmnunes@qui.uc.pt (C.M.N.); 321gilferreira@gmail.com (G.A.F.); kiarash.keyvan@yahoo.com (K.K.); rfausto@ci.uc.pt (R.F.); 3 Istanbul Kultur University, Faculty Sciences and Letters, Department of Physics, Bakirkoy, 34158 Istanbul, Turkey

**Keywords:** *C*-aminophenyl-nitrilimines, bond-shift isomers, matrix-isolation, IR spectroscopy, photochemistry, DFT calculations

## Abstract

The intriguing ability of *C*-phenyl-nitrilimine to co-exist as allenic and propargylic bond-shift isomers motivated us to investigate how substituents in the phenyl ring influence this behavior. Building on our previous work on the *meta*- and *para*-OH substitution, here we extended this investigation to explore the effect of the NH_2_ substitution. For this purpose, *C*-(4-aminophenyl)- and *C*-(3-aminophenyl)-nitrilimines were photogenerated in an argon matrix at 15 K by narrowband UV-light irradiation (λ = 230 nm) of 5-(4-aminophenyl)- and 5-(3-aminophenyl)-tetrazole, respectively. The produced nitrilimines were further photoisomerized to carbodiimides via 1*H*-diazirines by irradiations at longer wavelengths (λ = 380 or 330 nm). Combining IR spectroscopic measurements and DFT calculations, it was found that the *para*-NH_2_-substituted nitrilimine exists as a single isomeric structure with a predominant allenic character. In contrast, the *meta*-NH_2_-substituted nitrilimine co-exists as two bond-shift isomers characterized by propargylic and allenic structures. To gain further understanding of the effects of phenyl substitution on the bond-shift isomerism of the nitrilimine fragment, we compared geometric and charge distribution data derived from theoretical calculations performed for *C*-phenyl-nitrilimine with those performed for the derivatives resulting from NH_2_ (electron-donating group) and NO_2_ (electron-withdrawing group) phenyl substitutions.

## 1. Introduction

Nitrilimines (R′–CNN–R″), first reported by Huisgen and co-workers [1], constitute highly versatile intermediates in organic synthesis, particularly in regio- and stereoselective 1,3-cycloaddition reactions, yielding five-membered heterocycles [2,3,4,5,6,7]. Their utility spans across diverse disciplines, from general synthetic chemistry to bioorthogonal chemistry and materials science [6,8,9,10]. These 1,3-dipolar species are typically generated in situ by photolysis or pyrolysis of tetrazoles [11,12,13]. Due to the highly reactive nature of nitrilimines, matrix-isolation infrared spectroscopy, in conjugation with ab initio calculations, is one of the most effective methodologies for their characterization [3,14,15,16,17,18,19,20].

**Scheme 1:**
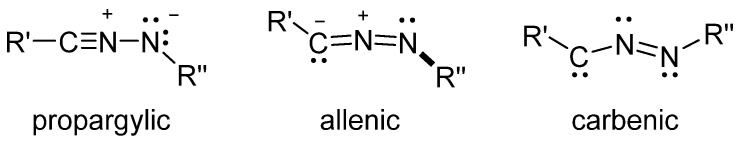
Idealized geometric structures of nitrilimines.

In general, nitrilimines are monoisomeric species and, depending on the R′ and R″ substituents, exhibit predominantly propargylic or allenic structures [3,16]. However, some nitrilimines with amino (R′ = NH_2_, R″ = H, or NH_2_), hydroxy (R′ = H or OH, R″ = OH), and fluoro (R′ = F, R″ = H, or F) substitutions have been shown to possess a significant carbenic character [19,21,22] (Scheme 1). Experimental identification of nitrilimines with distinct structural characteristics can be accomplished by analyzing the infrared frequency of the antisymmetric CNN stretching vibration [3,16,19,21]. Frequencies in the 2300–2150 cm^−1^ range indicate nitrilimines predominantly propargylic, possessing a CN triple bond, while frequencies below the 2150–2000 cm^−1^ range denote allenic-type nitrilimines with a cumulenic CNN moiety. Nitrilimines characterized by weak absorption peaks below 2000 cm^−1^ have a strong carbenic character.

**Scheme 2:**
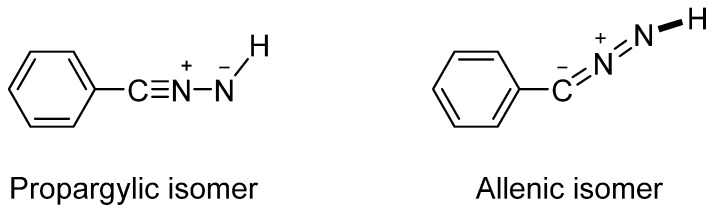
Bond-shift isomers of *C*-phenyl-nitrilimine.

A particularly puzzling behavior has been observed for *C*-phenyl-nitrilimine (R′ = Ph, R″ = H). This molecule was theoretically predicted to exhibit two minima on the potential energy surface: one characterized by an allenic-like structure and the other by a propargylic-like structure [16] (Scheme 2), representing genuine bond-shift isomers [23,24]. The experimental demonstration of this theoretically predicted behavior was achieved in our laboratory through the spectroscopic identification of these two isomeric forms under matrix-isolation conditions [18]. This finding prompted us to investigate how substituents on the phenyl ring affect the bond-shift isomerism of the nitrilimine fragment. Our subsequent study on *C*-hydroxyphenyl-nitrilimines showed that the OH phenyl substitution leads to different outcomes depending on its position [17]. When the OH group is in the *meta* position, the nitrilimine has structural features similar to the parent compound, displaying two bond-shift isomers with propargylic and allenic characters. In contrast, when the OH group is in the *para* position, only a single isomer with a strong allenic character was observed.

Inspired by these results, the present study investigates both experimentally and theoretically the effects of the NH₂ substitution at the *meta* and *para* positions on the bond-shift isomeric properties of *C*-phenyl-nitrilimine. These investigations involved the use of tunable narrowband UV light to generate *C*-aminophenyl-nitrilimines, from matrix-isolated 5-aminophenyl-tetrazoles, and the exploration of their photochemistry. Infrared spectroscopy, supported by DFT theoretical calculations, was employed to identify and characterize the photoproduced species. In addition, to gain deeper insights into the effects of the phenyl substitution on the bond-shift isomerism of the nitrilimine fragment, we expanded the theoretical studies to compare the influence of both electron-donating (NH_2_) and electron-withdrawing (NO_2_) groups in *ortho*, *meta*, and *para* positions relative to the unsubstituted *C*-phenyl-nitrilimine. This comprehensive approach enabled us to investigate both positional effects and the inherent characteristics of substituents.

## 2. Results and Discussion

### 2.1. Structure and Matrix-Isolated IR Spectra of 5-Aminophenyl-Tetrazoles

In this study, 5-(4-aminophenyl)-tetrazole will be denoted as **1p**, and 5-(3-aminophenyl)-tetrazole as **1m**, in accordance with the nomenclature previously used by us for the analogous hydroxy-substituted 5-phenyl-tetrazoles (letters **p** and **m** signify the *para* and *meta* positions of the substituent group in the phenyl ring relative to the tetrazole ring, respectively) [17]. Both molecules can exist in the 1*H* or 2*H* tautomeric forms (see Table 1), abbreviated here as **1p′/1m′** or **1p″/1m″**, respectively, which is also consistent with the terminology followed in our previous work [17]. However, it has been established that direct access to nitrilimines is only viable through the 2*H* tautomer [16,17,18,19,20,25]. Therefore, to theoretically evaluate the potential of compounds **1p** and **1m** as candidates for the photoproduction of the corresponding *C*-(4-aminophenyl)-nitrilimine **2p** and *C*-(3-aminophenyl)-nitrilimine **2m**, the former were energetically characterized at the B3LYP/6-311++G(d,p) level of theory and the obtained results are summarized in Table 1. Based on the Boltzmann populations estimated from the Gibbs energies at 380 K (temperature of the sublimated compounds before the matrix deposition, see Section 3), the **1p″**/**1p′** and **1m″**/**1m′** tautomeric ratios were determined to be 92/8 and 96/4, respectively, thus demonstrating the suitability of **1p** and **1m** as precursors of nitrilimines. The higher stability of the **1p″** or **1m″** isomers (2*H* tautomeric forms) compared to the **1p′** or **1m′** isomers (1*H* tautomeric forms) can be attributed to the increased electron delocalization within the tetrazole ring in the formers [26], which is reflected by the longer formal N=N and C=N double bonds and shorter formal N-N single bond (see caption of Table 1).

Molecule **1p″** does not display conformers. However, molecule **1m″** exhibits two stable conformations distinguished by *syn* (**s**) and *anti* (**a**) orientations of the tetrazole NH group relative to the phenyl NH_2_ group (see Table 1 for details). According to the calculations, **1m″-a** is the most stable conformer. The Gibbs energy of **1m″-s** relative to **1m″-a** is less than 1 kJ mol^−1^, resulting in a computed **1m″-a**/**1m″-s** ratio of 57/39 at 380 K (Table 1). Consequently, a mixture of **1m″-a** and **1m″-s** conformers should dominate the vapor phase prior to the Ar matrix deposition. Besides the gas-phase populations, evaluation of the energy barrier for the conversion of the higher (**1m″-s)** into the lower energy (**1m″-a**) conformer is also relevant for predicting the composition of the deposited Ar matrix [27,28,29,30]. The energy barrier for the conformational relaxation, obtained from the B3LYP potential energy profile (PES) for the internal rotation around the inter-rings C−C bond, was calculated to be 20 kJ mol^−1^ (Figure S1). This value is sufficiently large to allow efficient trapping of both conformers in an Ar matrix at 15 K [27,28,29,30].

**Figure 1:**
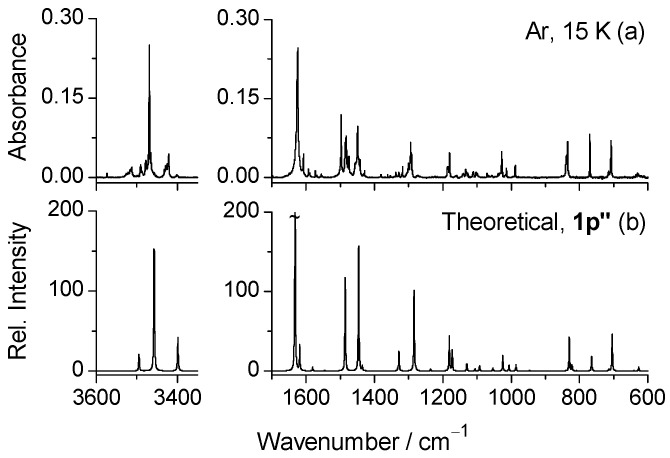
(**a**) Experimental IR spectrum of 5-(4-aminophenyl)-tetrazole **1p** in an Ar matrix at 15 K; (**b**) B3LYP/6-311++G(d,p) theoretical IR spectrum of **1p″**.

**Figure 2:**
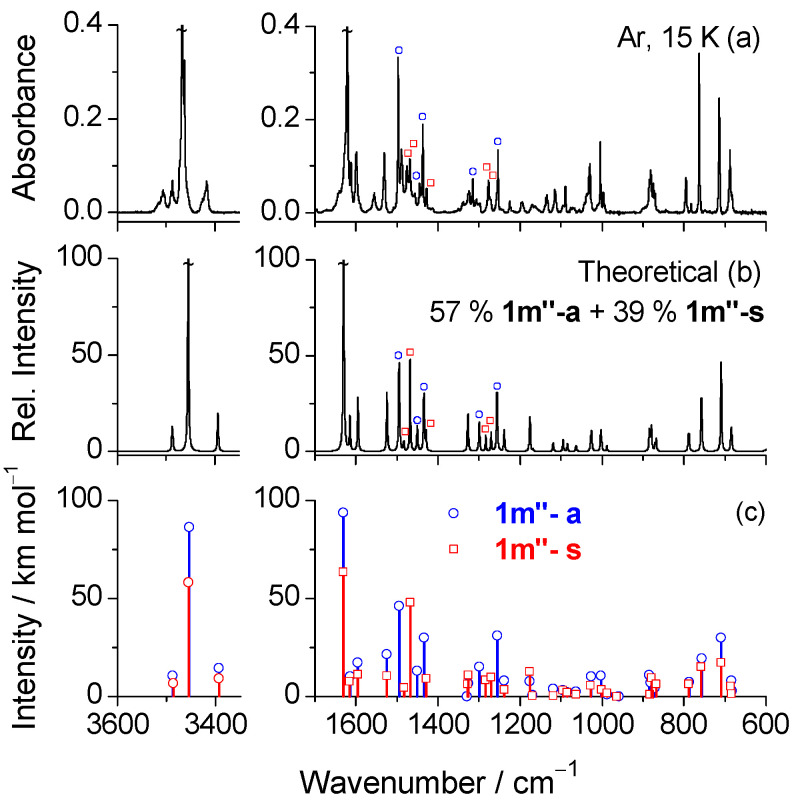
(**a**) Experimental IR spectrum of 5-(3-aminoyphenyl)-tetrazole **1m** in an Ar matrix at 15 K; (**b**) theoretical IR spectrum of **1m″** obtained as a sum of the B3LYP/6-311++G(d,p) spectra calculated for the **1m″-a** and **1m″-s** conformers with their intensities scaled by the respective gas-phase Boltzmann populations; (**c**) wavenumbers and IR intensities (both scaled) extracted from the B3LYP/6-311++G(d,p) harmonic vibrational calculations carried out for the two conformers.

Figure 1a and Figure 2a depict the experimental IR spectra of **1p** and **1m**, respectively, isolated in an Ar matrix at 15 K, accompanied by the theoretical IR spectra of **1p″** and **1m″**, shown in Figure 1b and Figure 2b. For **1m″**, the theoretical spectrum was derived by co-adding the B3LYP spectra calculated for the two predicted conformers (Figure 2c) with the intensities multiplied by their estimated gas-phase Boltzmann populations. Overall, there is a good correspondence between the experimental spectra of matrix-isolated **1p** and **1m** and the theoretical spectra computed for **1p″** and **1m″**, respectively. On the contrary, the experimental spectra are not well reproduced by the spectra computed for **1p′** or **1m′** (Figures S2 and S3). This is consistent with the theoretical predictions and demonstrates that there is an overwhelming predominance of the 2*H* tautomeric forms in the as-deposited Ar matrices.

Tables S1 and S2 present the proposed IR band assignments for **1p** and **1m**, respectively, which were based on the comparison of the experimental spectra with the computed vibrational data. The higher frequency region of the two experimental mid-IR spectra is characterized by a very strong band at 3469−3467 cm^−1^, which is characteristic of the tetrazole ν(NH) mode [26], together with two weaker bands at 3513 and 3421 cm^−1^ for **1p**, or at 3507 and 3417 cm^−1^ for **1m**, corresponding to the ν_as_(NH_2_) and ν_s_(NH_2_) modes, respectively. In the fingerprint region, the spectrum of **1p** is dominated by the bands at 1625 [δ(NH_2_)], 1498/1483 [δ(CNN); δ(NCN); δ(NH); ν(CN)_tz_], 1449 [ν(CC)_ph-tz_; δ(NCN)], and 1294 [ν(CC_NH2_)] cm^−1^, while the spectrum of **1m** is dominated by the bands at 1621 [δ(NH_2_)], 1498 [δ(CNN); ν(CN)_tz_; δ(NH)], 763 [γ(C)_tz_], and 714 [γ(N)_tz_] cm^−1^. Even though the spectra calculated for the two **1m″** conformers are very similar (as shown in Figure 2c), a detailed spectral analysis allows the identification of five experimental bands specific to each of them. Spectral features of **1m″-a** are observed at 1498, 1445, 1437, 1315, and 1253 cm^−1^ (highlighted by blue circles in Figure 2a), while spectral signatures of **1m″-s** are found at 1476, 1469, 1428, 1277, and 1272 cm^−1^ (indicated by red squares in Figure 2a).

### 2.2. Photogeneration and Spectroscopic Characterization of C-(4-Aminophenyl)-Nitrilimine

**Figure 3:**
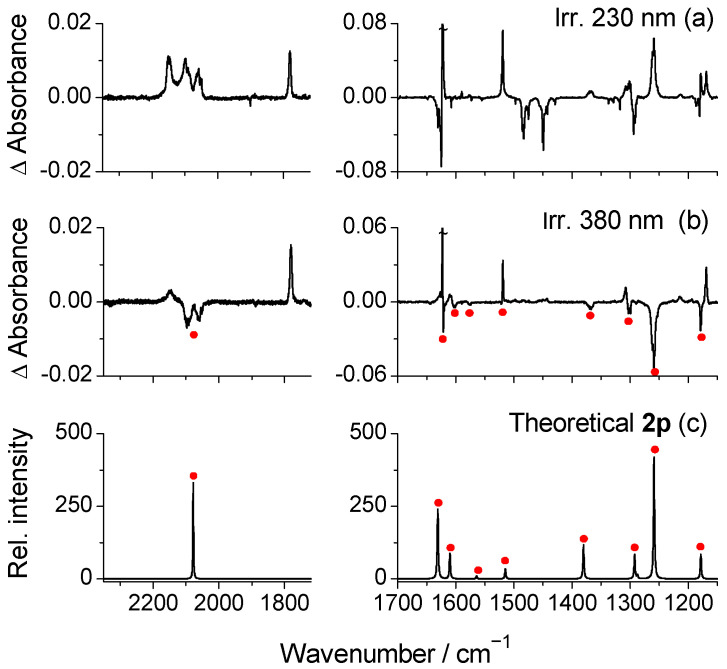
Experimental difference IR spectra (2300−1100 cm^−1^) showing changes (**a**) after irradiation of matrix-isolated **1p** (Ar, 15 K) at λ = 230 nm for ~1 min and (**b**) after subsequent ~26 min of irradiation at λ = 380 nm (the negative bands labeled by red circles are due to the consumed photoproduct assigned to **2p**); (**c**) theoretical IR spectrum of the most stable conformer of *C*-(4-aminophenyl)-nitrilimine **2p** (**2p-a**) calculated at the B3LYP/6-311++G(d,p) level (see Table S3 for details).

Phototransformations in **1p** isolated in solid Ar at 15 K were triggered by performing in situ irradiations with narrowband light at λ = 230 nm. This wavelength was chosen because of its demonstrated effectiveness in inducing photoreactions in analogous matrix-isolated molecules [17,18,19] and due to its proximity to the wavelength of maximum absorption in the UV spectrum of 5-phenyl-tetrazole dissolved in ethanol (λ_max_ = 229.4 nm) [31]. The spectral changes originated by the UV irradiations are depicted in the difference spectrum presented in Figure 3a for the 2300–1100 cm^−1^ range and in Figure S4a for the 3600–3200 cm^−1^ range. The negative bands indicate the consumption of **1p**, while the positive ones denote the appearance of photoproducts. Focusing on the bands emerging in the 2300–1700 cm^−1^ region, the doublet at 2098/2059 cm^−1^ appears shortly after 10 s of irradiation but ceases to grow for longer irradiation times. In contrast, the other two bands, observed at 2150 cm^−1^ and 1781 cm^−1^, continue to grow (see Figure S5 for details). These findings suggest that the first band should correspond to a primary photoproduct, whereas the remaining two should be associated with secondary photoproducts.

Considering the typical photochemical behavior reported for 2*H*-tetrazoles [11,32,33], it is highly plausible that the primary photoproduct would correspond to *C*-(4-aminophenyl)-nitrilimine **2p**, which is produced by extrusion of molecular nitrogen from 5-(4-aminophenyl)-tetrazole **1p**. To confirm this hypothesis, we have conducted subsequent irradiations at a longer wavelength (λ = 380 nm) with the aim of promoting the consumption of putative **2p** without affecting **1p**. The identification of **2p** was definitively confirmed by the excellent agreement of the negative part of the difference IR spectrum obtained by subtracting the spectrum recorded before the irradiation at λ = 380 nm from that recorded after the irradiation at this wavelength, with the B3LYP/6-311++G(d,p) theoretical spectrum of this species, as depicted in Figure 3b,c (and additionally in Figure S4). A detailed assignment of the IR spectrum of **2p** is provided in Table 2. Its most characteristic spectral feature is the doublet band at 2098/2059 cm^−1^, which is theoretically predicted at 2078 cm^−1^ and is ascribed to the antisymmetric stretching vibration of the CNN fragment [ν_as_(CNN)]. The placement of this band within the range of 2100–2000 cm^−1^ strongly suggests that the photogenerated nitrilimine adopts an allenic-type structure [3,16]. Further elaboration on this topic will be provided later.

**Figure 4:**
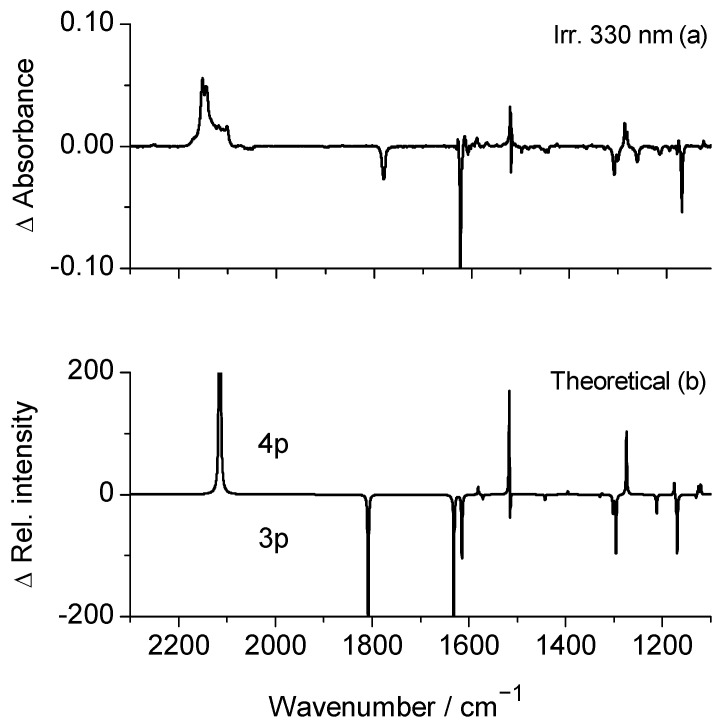
(**a**) Experimental difference IR spectrum showing changes after 15 min of irradiation at λ = 330 nm subsequently to the consumption of **2p** at 380 nm (Figure 3). The negative bands are due to the consumed 4-aminophenyl-1*H*-diazirine **3p** while the positive ones are assigned to the photogenerated 4-aminophenyl-carbodiimide **4p**. (**b**) B3LYP/6-311++G(d,p) theoretical difference spectrum obtained as the spectrum of the most stable conformer of **4p** minus the spectrum of the most stable conformer of **3p** (see Table S4 for details).

**Scheme 3:**
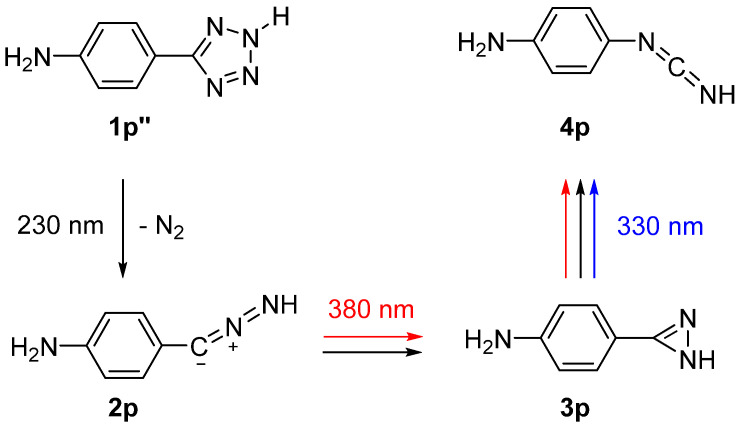
Summary of the UV-induced photochemistry of the 2*H*-tautomeric form of 5-(4-aminophenyl)-tetrazole **1p″** (Ar, 15 K): generation of *C*-(4-aminophenyl)-nitrilimine **2p** and its subsequent photoisomerization to 4-aminophenyl-1*H*-diazirine **3p** and 4-aminophenyl-carbodiimide **4p**. Black, red and blue arrows represent the phototransformations at 230, 380 and 330 nm, respectively.

Concomitantly with the decrease in the bands assigned to **2p**, other bands that emerged upon the excitations at λ = 230 nm, including those at 2150 cm^−1^ and 1781 cm^−1^, are intensified during the irradiations at λ = 380 nm (see Figure 3b). This observation demonstrates that the corresponding photoproducts are generated with the intermediacy of **2p**. Building upon existing knowledge of the photochemistry of nitrilimines, these photoproducts are most likely 4-aminophenyl-1*H*-diazirine **3p** and 4-aminophenyl-carbodiimide **4p** [3,17,18,19,34,35]. Both species could be easily identified through the outcomes of additional irradiations carried out at λ = 330 nm (after the consumption of **2p**). The comparison of the difference spectrum resulting from these irradiations (Figure 4a) with the calculated difference spectrum **4p**–**3p** (Figure 4b) demonstrates clearly that the negative bands are assigned to **3p** while the positive ones are assigned to **4p**, i.e., **3p** photorearranges to **4p** [16,20]. The main absorption of **3p** is that identified at 1781 cm^−1^ (predicted at 1808 cm^−1^), which is particularly characteristic of 1*H*-azirines [17,18,20,36] and is assigned to the three-membered ring ν(C=N) vibration. Regarding **4p**, its most prominent band is identified at 2150 cm^−1^ (predicted at 2115 cm^−1^), which is particularly characteristic of carbodiimides and is assigned to the ν_as_(NCN) vibration [16,17,18]. In summary, our results reveal that once **2p** is generated from **1p** by N_2_ elimination, it photoisomerizes to yield **4p** via **3p** (see Scheme 3). This reasoning agrees with the mechanism of reactivity that has been established in previous photochemical studies carried out for similar systems [17,18].

### 2.3. Photogeneration and Spectroscopic Characterization of C-(3-Aminophenyl)-Nitrilimine

**Figure 5:**
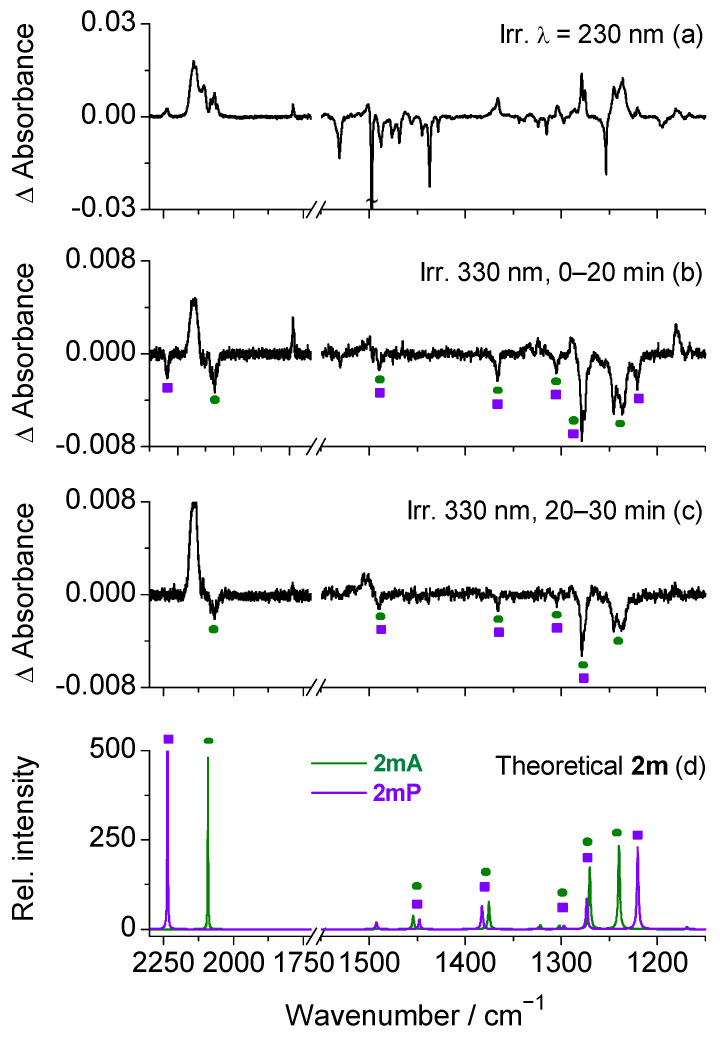
Experimental difference IR spectra (2300−1100 cm^−1^) showing changes (**a**) after irradiation of matrix-isolated **1m** (Ar, 15 K) at λ = 230 nm and (**b,c**) after subsequent irradiations at λ = 330 nm (the negative bands labeled by green circles and violet squares are due to the consumed photoproducts assigned to **2mA** and **2mP**, respectively); (**d**) theoretical IR spectra of most stable conformers of **2mA** (green) and **2mP** (violet) calculated at the B3LYP/6-311++G(d,p) level (see Table S3 for details).

**Figure 6:**
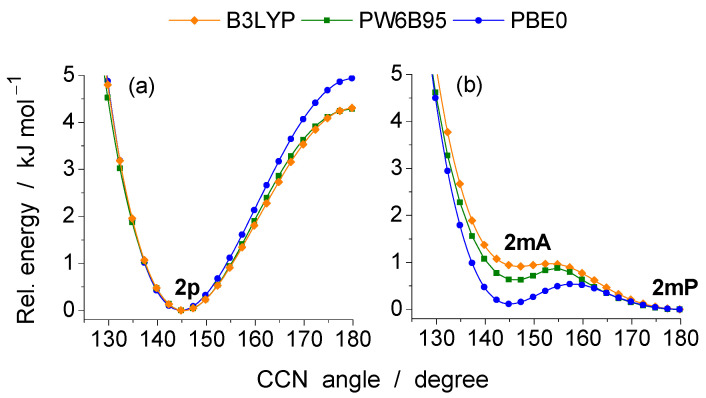
Relaxed potential energy scan as function of the CCN angle for (**a**) *C*-(4-aminophenyl)-nitrilimine **2p** and (**b**) *C*-(3-aminophenyl)-nitrilimine **2m**. The scans were computed using the B3LYP (blue), PW6B95 (green), and PBE0 (orange) DFT functionals with the 6-311++G(d,p) basis set. Along the scans, the value of the CCN angle was incrementally fixed in steps of 2.5 degrees, and all other geometrical parameters were fully optimized.

Exposing matrix-isolated **1m** to narrowband UV light tuned at λ = 230 nm resulted in the appearance of new bands at 2067 (triplet), 1787, and 2142/2106 cm^−1^ within the 2300–1700 cm^−1^ region (see Figure 5a and Figure S5). These spectral positions closely match those observed during the photolysis of **1p**, indicating the formation of analogous photoproducts: *C*-(3-aminophenyl)-nitrilimine **2m**, 3-aminophenyl-1*H*-diazirine **3m**, and 4-aminophenyl-carbodiimine **4m**. The only significative difference from the photochemical behavior of **1p** is the emergence of an additional band at 2239 cm^−1^, which displays an identical growth pattern to the band at ~2067 cm^−1^ (Figure S5), suggesting that it is also assigned to a primary photoproduct. This IR band falls within a spectral region where propargylic nitrilimines typically absorb [3,16,17,18]. Hence, it is most likely that both allenic and propargylic forms of **2m**, designated as **2mA** and **2mP**, respectively, are generated from **1m**, similar to the photochemistry observed for the 5-phenyl-tetrazole [18] and 5-(3-hydroxyphenyl)-tetrazole [17]. This interpretation is also supported by the computed PES as a function of the CNN bond angle, which reveals the existence of two separate energy minima for **2m** but only a single minimum for **2p** (Figure 6).

**Scheme 4:**
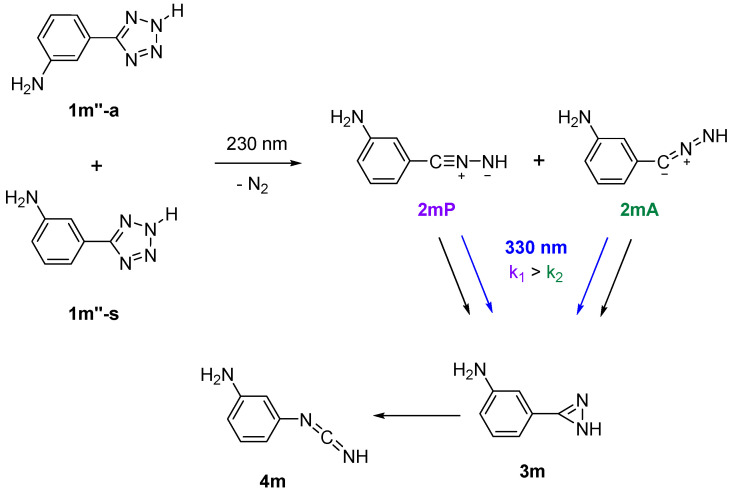
Summary of the UV-induced photochemistry of the 2*H* tautomeric form of 5-(3-aminophenyl)-tetrazole **1m″** (Ar, 15 K): generation of the propargylic (**2mP**) and allenic (**2mA**) bond-shift isomers of *C*-(3-aminophenyl)-nitrilimine **2m** and their subsequent photoisomerization to 3-aminophenyl-1*H*-diazirine **3m** and 3-aminophenyl-carbodiimide **4m** with distinct kinetics. Black and blue arrows represent the phototransformations at 230 and 330 nm, respectively.

Experimental evidence confirming the co-existence of the two bond-shift isomers was obtained through subsequent irradiations at λ = 330 nm. In the first 20 min of irradiation, several bands that had appeared after the initial irradiation of the sample at λ = 230 nm decreased in intensity (Figure 5b). After an additional 10 min of irradiation, some of these bands were completely depleted, while others continued to diminish (Figure 5c). This allowed the distinction of two species based on their different kinetics of consumption. From the comparison of the negative parts of the two obtained experimental difference IR spectra (Figure 5b,c) with the calculated IR spectra of **2mA** and **2mP** (Figure 5d), the following can be concluded: (i) the bands centered at 2238 and 1221 cm^−1^, corresponding to the species that experienced a more rapid consumption, match the ν_as_(CNN) and δ(NH) modes predicted for **2mP** at 2236 and 1220 cm^−1^, respectively; (ii) the bands at 2067 and 1246/1236 cm^−1^, ascribed to the species that experienced a slower consumption, are well reproduced by the ν_as_(CNN) and δ(NH) modes theoretically predicted for **2mA** at 2091 and 1240 cm^−1^. These results clearly confirm the in situ generation of both propargylic- and allenic-type isomers of **2m** upon UV irradiation of matrix-isolated **1m″** (Scheme 4). An assignment of the IR spectra of **2mA** and **2mP** is given in Table 3. The faster kinetics of consumption of **2mP** (*k*_1_)compared to **2mA** (*k*_2_) under UV irradiation at λ = 330 nm is consistent with the significantly higher absorptivity of the former relative to the latter, as deduced from the comparison of the TD-DFT UV spectra for the two isomers (Figure S6). The set of IR bands that increase upon the irradiation at λ = 330 nm, due to the consumption of **2mP** and **2mA,** are attributed to **3m** and **4m**, with the most characteristic spectral features being identified at 1787 cm^−1^ for **3m** [ν(C=N)] and at 2067 cm^−1^ for **4m** [ν_as_(NCN)].

### 2.4. Stability of the Bond-Shift Isomers and Their Attempted Interconversion

As shown in Figure 6, the energy barrier separating **2mP** and **2mA** is very small (less than 1 kJ mol^−1^). Additionally, the energy difference between the two isomers is also very small (Table S3). According to these data, it can be hypothesized that the co-existence of these two isomers in the low-temperature Ar matrix is due to the fact that they are in equilibrium. However, our experimental observations do not support this hypothesis. In fact, after subjecting the sample to 20 min of UV irradiation at λ = 330 nm, resulting in the selective depletion of **2mP**, we monitored the sample for several hours (13 h) in dark conditions (a metal plate was placed between the source of the spectrometer and the sample) to assess whether this isomer could be regenerated at the expense of **2mA**. However, no such regeneration was observed, definitively showing that the two isomers exist independently in the cryogenic matrix.

**Figure 7:**
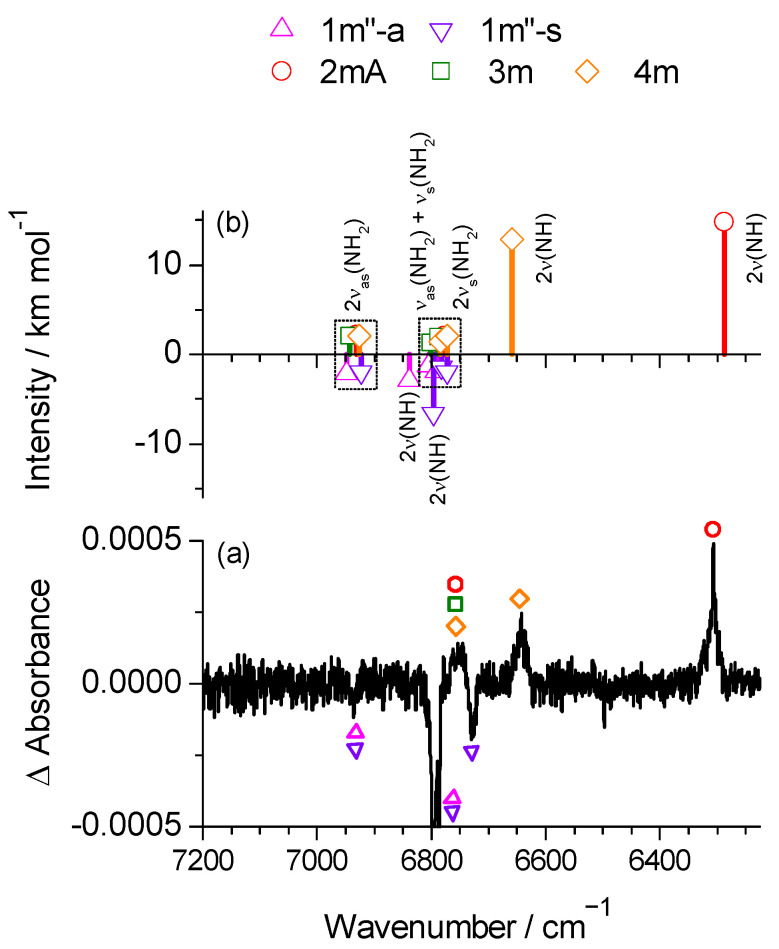
(**a**) Selected region of a difference near-IR spectrum (7200–6050 cm^−1^) showing changes after 20 min UV irradiation of **1m″** at λ = 230 nm, followed by subsequent 20 min of irradiation at λ = 330 nm. Positive bands are assigned to **2mA**, **3m,** and **4m** species (**2mP** was not identified as it was consumed during the first 20 min of irradiation at λ = 330 nm, Figure 5), while the negative ones are assigned to **1m″-a** and **1m″-s**. (**b**) B3LYP/6-311++G(d,p) anharmonic wavenumbers and IR intensities (both unscaled) calculated for the first overtones of the NH stretching vibration [2ν(NH)], first overtones of the antisymmetric [2ν_as_(NH_2_)] and symmetric [2ν_s_(NH_2_)] stretching vibrations, and ν_as_(NH_2_) + ν_s_(NH_2_) combination modes (see Table S6 for details).

Having established that **2mA** and **2mP** are not in equilibrium in the Ar matrix, we attempted their interconversion by vibrational excitation [36,37,38] with narrowband NIR light produced by a laser-OPO source or with broadband NIR/mid-IR light emitted from the IR spectrometer source. To determine the specific frequencies of vibrational modes available for performing selective NIR vibrational excitation, two spectra were recorded in the 7500–4000 cm^−1^ region: one immediately after the deposition of **1m** in the Ar matrix and another after a series of UV irradiations at λ = 230 nm, followed by irradiations at λ = 330 nm to deplete **2mP** while retaining **2mA** in the matrix. The resulting NIR difference spectrum was compared with B3LYP/6-311++G(d,p) anharmonic vibrational calculations for the precursor and photoproducts (see Figure 7 and Table S6).

This spectral comparison allowed us to unequivocally identify the band corresponding to the first overtone of the ν(NH) stretching vibration of **2mA** at 6307 cm^−1^. We also tried to identify the 2ν_as_(NH_2_) and 2ν_s_(NH_2_) overtones, as well as the ν_as_(NH_2_) + ν_s_(NH_2_) combination band corresponding to this species. However, as shown in Figure 7, these bands overlap with those of the precursor and other photoproducts, making it difficult to pinpoint the exact frequencies suitable for laser irradiations. Irradiating the matrix for 20 min with the laser tuned to 6307 cm^−1^ did not produce any spectral changes indicating the **2mA** → **2mP** conversion. The exposure of the sample to the broadband NIR/mid-IR radiation emitted by the spectrometer source for approximately 50 h also did not result in any detectable interconversion. Although unexpected, considering the theoretical data, these results are in line with previous findings for *C*-phenyl-nitrilimine [18] and *C*-(3-hydroxyphenyl)-nitrilimine [17]. For both molecules, two bond-shift isomers (allenic and propargylic) were also found to co-exist in an Ar matrix after photolysis of the corresponding tetrazole precursors, and in both cases, their interconversion was not achieved either by annealing experiments (for *C*-phenyl-nitrilimine) or by selective NIR excitation of the OH stretching overtones (for *C*-(3-hydroxyphenyl)-nitrilimine). Two factors may help to explain this observed behavior in the bond-shift isomers of the three phenyl-nitrilimines. Firstly, intrinsic limitations of theoretical methods that might hinder accurate calculation of the PES for the interconversion between the two bond-shift isomers. Secondly, the matrix-environment may lead to an increase in the energy barrier separating the two isomers, making their interconversion more difficult, as it has been reported in some other cryogenic matrix-isolation experiments involving systems with conformational isomers separated by very small energy barriers [24,39,40,41,42,43].

### 2.5. Effect of the Position and Nature of the Phenyl Substitution on the Structure of C-Phenyl-Nitrilimines

According to the experimental results, the structure of photogenerated *C*-aminophenyl-nitrilimines varies with the positioning of the NH_2_ group relative to the CNN fragment. For the *para*-NH_2_ substitution (**2p**), only one isomeric form is identified. Conversely, for the *meta*-NH_2_ substitution (**2m**), two distinct isomeric forms are observed: **2mA** and **2mP**. This is consistent with the PES calculated for the two nitrilimines at different levels, as depicted in Figure 6. Specifically, a single minimum is predicted for **2p**, while two minima are predicted for **2m**. Based on the wavenumbers corresponding to the ν_as_(CNN) band—2098/2057 cm^−1^ for **2p**, 2067 cm^−1^ for **2mA**, and 2239 cm^−1^ for **2mP**—it can be inferred that **2p** and **2mA** are predominantly allenic, while **2mP** is predominantly propargylic. This is corroborated by the geometric data extracted from the B3LYP/cc-pVTZ fully optimized geometries, as detailed in Table 4. Both **2p** and **2mA** have a non-planar structure (C_R_C_N_N_H_H dihedral = 98–99º) and a bent CCN framework (C_R_C_N_N_C_ angle = 142–143º). Conversely, **2mP** has a more planar structure (C_R_C_N_N_H_H dihedral = 161º), together with a much straighter CCN framework (C_R_C_N_N_C_ angle = 178º). Additionally, the CN bond is shorter and the NN bond is longer in **2mP** compared to **2mA** and **2p**. The experimental and theoretical results obtained for **2p** and **2m** align with the results of previous studies on analogous OH-substituted phenyl nitrilimines [17]. From these findings, a general conclusion can be drawn: electron-donor substituents located at the *para* position relative to the CNN fragment promote an allenic-type structure, whereas *meta* substitution stabilizes both allenic and propargylic structures.

To deepen our understanding of how phenyl substitution affects the structure of the CNN fragment, we extended our theoretical study to include a derivative with an electron-withdrawing substituent (NO_2_) and also considered the *ortho* position for both NH_2_ and NO_2_ substitutions. The PES computed for these newly explored molecules are depicted in Figure S7, and the geometric data extracted from the B3LYP/cc-pVTZ fully optimized geometries of the respective minima are provided in Table 4. The results for the *meta*-NO_2_ substitution closely resemble those obtained for the *meta*-NH_2_ substitution, where both the allenic and propargylic structures are potentially stable minima, though the corresponding PES is even flatter. Moreover, the corresponding geometric parameters exhibit similar trends comparing the *meta*-NO_2_ with the *meta*-NH_2_ substitution. Regarding the *ortho* and *para* substitutions, there is a notable difference between the two substituents. For NH_2_, both substitutions stabilize allenic-type isomers exhibiting identical geometric parameters, whereas for NO_2_, they stabilize propargylic-type isomers that also share similar geometric features (Figure S7 and Table 4).

To better comprehend the influence of the phenyl substitution on the structure of the nitrilimine fragment, we have calculated the charge distributions in the NH_2_- and NO_2_-substituted *C*-phenyl-nitrilimines, as well as in the unsubstituted molecule (used as reference), and the obtained results are included in Table 4. The Hirshfeld population analysis [44] was used for this purpose because of its proven accuracy in estimating atomic charges of organic molecules [45] and its ability to provide a comprehensive description of both reactivity and regioselectivity in electrophilic aromatic substitutions [46,47,48]. For *C*-phenyl-nitrilimine, the interplay of resonance and inductive effects originated by the phenyl ring leads to the stabilization of both allenic and propargylic forms [18]. The allenic form is characterized by a negative charge at the nitrilimine carbon atom (C_N_) and a positive charge at the adjacent nitrogen atom (N_C_), whereas in the propargylic form, N_C_ carries a positive charge while the terminal nitrogen (N_H_) bears a negative charge (see Scheme 1). The *meta* substitution reveals that regardless of the substituent’s nature, the charge distribution within the nitrile imine fragment is not much affected with respect to that of the analogous unsubstituted isomers. This explains why both allenic and propargylic forms remain minima on the respective PESs. Rather different results are obtained for the *ortho* and *para* substitutions, depending on the substituent nature. For the NH_2_ derivatives, the ring carbon atom attached to the CNN group (C_R_) becomes significantly more negatively charged compared to the unsubstituted molecule. This is consistent with the electron-donating nature of this substituent, which increases the electron density at the ring carbons in the *ortho* and *para* positions. According to the results presented in Table 4, the increased electron charge in C_R_ is also partly delocalized to C_N_, which also becomes negatively charged. Such an increase in the negative charge at C_N_ favors the allenic-type structure and consequently disfavors the propargylic one. Regarding the NO_2_ derivatives, C_R_ becomes significantly more positively charged as compared to the unsubstituted molecule. This is also consistent with the electron-withdrawing nature of this group, which decreases the electron charge of the ring carbon in *ortho* or *para* positions with respect to the substituent. This electron withdrawal is also extended to the C_N_ atom, which results in the destabilization of the allenic form and consequent stabilization of the propargylic one.

## 3. Methods

### 3.1. Experimental Methods

Commercial samples of 5-(4-aminophenyl)-tetrazole (**1p**, Alfa Aesar, 97% purity) and 5-(3-aminophenyl)-tetrazole (**1m**, Alfa Aesar, 97% purity) were sublimated at ~380 K inside a miniature glass oven connected to the vacuum chamber of a closed-cycle helium cryostat (APD Cryogenics, with a DE-202A expander), through a stainless steel needle valve. Before cooling the cryostat, a freeze–pump–thaw procedure was performed to purify the sample from volatile impurities. The matrices were prepared by co-deposition of vapors resulting from the sublimation of the compounds with an excess of argon (Air Liquid, N60, purity 99.999%) onto a CsI optical substrate, cooled down to 15 K. The temperature of the CsI window was measured directly at the sample holder using a silicon diode sensor connected to a digital controller (Scientific Instruments, West Palm Beach, FL, USA, model 9650-1). Spectra in the mid-IR region (4000–400 cm^−1^) were recorded with a resolution of 0.5 cm^−1^ employing a Thermo Nicolet 6700 FTIR spectrometer, equipped with a cadmium telluride (MCT-B) detector and a potassium bromide (KBr) beam splitter. Spectra in the NIR region (7500–4000 cm^−1^) were also recorded with a resolution of 1 cm^−1^, using the same spectrometer and an Indium Gallium Arsenide (InGaAs) detector with a CaF_2_ beam splitter. The spectrometer underwent continuous purging with a stream of dry, CO_2_-filtered air to eliminate atmospheric water and CO_2_ vapors. The prepared matrixes were then irradiated through the outer quartz window of the cryostat by applying narrowband UV radiation (fwhm = 0.2 cm^−1^) provided by the frequency-doubled signal beam of a Quanta-Ray MOPO-SL optical parametric oscillator (OPO), pumped with a pulsed Nd:YAG laser pro 230 from Spectra-Physics (repetition rate = 10 Hz, pulse energy ~1–3 mJ, duration = 10 ns). The UV irradiations were performed at λ = 230, 308, 330, and 380 nm, with different exposure times.

### 3.2. Computational Methods

All calculations were carried out using the Gaussian 16 (Revision B.01) program package [49]. The geometries of the experimentally studied molecules were fully optimized at the B3LYP/6-311++G(d,p) [50,51,52] level of theory, followed by harmonic vibrational calculations at the same level. The B3LYP/6-311++G(d,p) vibrational data were then used to assist the spectral interpretation. To account for the incomplete treatment of electron correlation, basis set limitations, and neglected anharmonic effects, the harmonic vibrational frequencies were multiplied by 0.980 for the region below 2000 cm^−1^ and 0.950 for the region above 2000 cm^−1^ [17]. Theoretical IR spectra were obtained from the vibrational data by convoluting the peaks with Lorentzian functions (fwhm = 2 cm^−1^) centered at the scaled wavenumbers and peak heights matching the calculated IR intensities. An approximate description of the vibrational modes was based on the results provided by the “vibAnalysis” software (version 1.2.2) [53], supported by ChemCraft animation of the vibrations [54]. Anharmonic vibrational calculations were also carried out at B3LYP/6-311++G(d,p) level using the fully automated second-order vibrational perturbative approach developed by Barone and co-workers [55,56]. For some of the studied molecules, theoretical absorption UV-spectra was simulated from time-dependent density functional theory calculations (TD-DFT) [57,58] performed at the B3LYP/6-311++G(d,p) level. The Cartesian coordinates extracted from full geometry optimizations carried out for all experimentally studied molecules are provided as Supplementary Materials.

Additional calculations were performed for *C*-phenyl-nitrilimine, as well as for the NH_2_- and NO_2_-substituted *C*-phenyl-nitrilimines in *ortho*, *meta*, and *para* positions. This included relaxed potential energy scans as a function of the nitrilimine CNN angle at the B3LYP/6-311++G(d,p), PBE0 [59]/6-311++G(d,p), and PW6B95 [60]/6-311++G(d,p) levels (only for the substituted *C*-phenyl-nitrilimines) and full geometry optimizations of the identified minima (plus vibrational calculations) at the B3LYP/cc-pVTZ level. This basis set was chosen for consistency with calculations previously performed on analogous molecules [17]. The outcomes of the optimizations at this level served to obtain geometric and charge distribution data for nitrilimines.

## 4. Conclusions

The effect of substitution in the phenyl ring on the structure of *C*-phenyl-nitrilimines has been explored in this study by considering the *para-* and *meta*-NH_2_-substituted compounds (*C*-(4-aminophenyl)- and *C*-(3-aminophenyl)-nitrilimines, respectively), which were experimentally studied by matrix-isolation spectroscopy and quantum chemical calculations. The comprehensive results obtained for these compounds, which bear a π electron-donating substituent, were complemented by computational studies performed on the *ortho*-NH_2_ derivative, as well as on the *ortho-*, *meta-*, and *para-*NO_2_-substituted analogous *C*-phenyl-nitrilimines, which bear a π electron-withdrawing substituent. Our previous data for the parent compound and the OH-substituted *C*-phenyl-nitrilimines [16,17,18] were also considered for the elucidation of the effects of the nature of the substituents on the structure of the *C*-phenyl-nitrilimines, in particular the existence of different bond-shift isomers.

The amino-substituted *C*-phenyl-nitrilimines were photogenerated in situ, in an argon matrix at 15 K, by excitation at λ = 230 nm of their tetrazole precursors (5-(4-aminophenyl)- and 5-(3-aminophenyl)-tetrazole, respectively). The UV-induced formation of the nitrilimines was confirmed by additional photochemistry experiments where these species were subsequently isomerized to carbodiimides via 1*H*-diazirines by irradiations at longer wavelengths (λ = 380–308 nm).

The combined results of the experiments and computations allowed us to conclude that, while the *para*-NH_2_ substitution stabilizes a single isomeric structure of the predominant allenic type, the *meta*-NH_2_ substitution leads to the co-existence of both propargylic- and allenic-type bond-shift isomers, a trend that we have also observed before for the analogous OH-substituted *C*-phenyl-nitrilimines [17].

The calculations performed on the whole set of molecules mentioned above, in particular the obtained equilibrium geometries, potential energy surfaces landscapes, and Hirshfeld charges, permitted attaining a comprehensive understanding of the effects of phenyl substitution on the bond-shift isomerism in *C*-phenyl nitrilimines. Parent *C*-phenyl-nitrilimine exists in both allenic and propargylic forms [18]. Regardless of the substituent’s nature, *meta* substitution (either electron donating or withdrawing) does not affect substantially the electronic features within the nitrilimine fragment, so *meta* substitution preserves the co-existence of the allenic and propargylic forms observed for the parent compound. On the other hand, *ortho* and *para* substitutions considerably change the electronic structure of the nitrilimine moiety. For electron donating substitutions (as in the case of the OH and NH_2_ derivatives), an increase in the negative charge at C_N_ takes place, which favors the allenic-type isomer that becomes the single stable isomeric form, while in the case of electron-withdrawing substitutions (as for the NO_2_ derivatives), the C_N_ atom becomes more positive, destabilizing the allenic form and leading to a consequent stabilization of the propargylic one, which then became the unique stable bond-shift isomeric form.

As a final remark, this study underscores the significant influence of substituent position and nature on the electronic structure and isomerism of *C*-phenyl-nitrilimines. The findings provide valuable insights for future research and potential applications involving substituted nitrilimines, particularly in designing compounds with specific electronic and structural properties.

## Figures and Tables

**Table 1:** Structures, relative Gibbs energies at 380 K (Δ*G*, in kJ mol^−1^) computed at the B3LYP/6-311++G(d,p) level of theory and the respective Boltzmann populations at 380 K (Pop., in %) for the tautomers and conformers of 5-(4-aminophenyl)-tetrazole **1p** and 5-(3-aminophenyl)-tetrazole **1m** ^a^.

5-(4-aminophenyl)-tetrazole **1p**				
Isomer	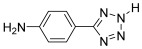		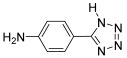	
	**1p″**		**1p′**	
Δ*G* (380 K)	0.00		6.13	
Pop.	92.2		7.8	
5-(3-aminophenyl)-tetrazole **1m**				
Isomer	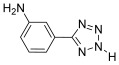	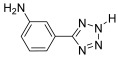	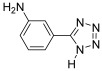	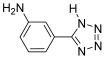
	**1m″-a**	**1m″-s**	**1m′-a**	**1m′-s**
Δ*G* (380 K)	0.00	0.97	6.91	10.79
Pop.	57.1	38.6	3.5	0.7

^a^ In the isomers designations, **1p′/1m′** or **1p″/1m″** correspond to the 1*H* or 2*H* tautomeric forms, respectively. In the case of **1m**, the letter after the hyphen denotes the relative orientation of the NH and NH_2_ groups (**a** = *anti*; **s** = *syn*). Mean bond lengths in the tetrazole ring extracted from the full geometry optimizations carried out for **1p″**, **1m″-a**, and **1m″-s** (2*H* tautomeric forms): C=N, 1.333 ± 0.001 Å; N=N, 1.303 ± 0.001 Å; N-N, 1.326 ± 0.001 Å. Mean bond lengths in the tetrazole ring extracted from the full geometry optimizations carried out for the **1p′**, **1m′-a** and **1m′-s** (1*H* tautomeric forms): C=N, 1.324 ± 0.001 Å; N=N, 1.288 ± 0.001 Å; N-N, 1.354 ± 0.002 Å.

**Table 2:** Experimental (Ar, 15 K) and B3LYP/6-311++G(d,p) computed infrared absorptions for *C*-(4-aminophenyl)-nitrilimine **2p** and approximate vibrational assignments.

Ar Matrix ^a^	Computed ^b^		Vibrational Assignment ^c^
	*ν̃*	*A^th^*	
3523	3502.6	24.3	ν_as_(NH_2_)
3432/3428	3404.5	57.1	ν_s_(NH_2_)
n.o.	3329.8	1.2	ν(NH)
2098/2059	2077.9	333.3	ν_as_(CNN)
1621	1631.0	241.1	δ(NH_2_)
1603	1610.1	88.5	ν(CC)_ph_
1576	1564.4	9.8	ν(CC)_ph_
1522	1515.0	34.7	ν(CN_A_) − ν(CC_N_); ν(CC)_ph_; δ(CH)
1367	1380.4	117.5	ν(NN) − ν(CC_N_)
1300	1292.5	83.5	ν(CN_A_); ν(CC)_ph_
1290	1287.5	12.4	ν(CC)_ph_
1259	1258.9	423.2	δ(NH)
1179	1178.5	84.9	δ(CH)
1124	1127.0	14.8	δ(CH)
1098	1098.5	28.9	ν(NN) + ν(CC_N_)
n.o.	1046.9	7.4	ρ(NH_2_)
831	829.8	15.5	ν(CC)_ph_
831	823.4	45.0	γ(CH)_ph_; γ(C)_ph_

^a^ Experimental wavenumbers are given in cm^−1^. Only the bands in the 3600–3200 cm^−1^ and 2300–800 cm^−1^ regions are included. Abbreviations: n.o. = not observed. ^b^ Data extracted from the vibrational calculations carried out for the B3LYP/6-311++G(d,p) lowest energy conformer of **2p** (**2p-a**); see Table S3 for details. Calculated harmonic wavenumbers were multiplied by 0.950 (above 2000 cm^−1^) or 0.980 (below 2000 cm^−1^). Vibrations predicted with very low intensities and lacking counterparts in the experimental spectrum were omitted. ^c^ Vibrational assignment was based on the results provided by the “vibAnalysis” software, supported by ChemCraft animation of the vibrations. Abbreviations: ν, stretching; δ, in-plane bending; γ, out-of-plane bending; ρ, rocking; ph, phenyl ring; s, symmetric; as, antisymmetric; C_N_ refers to the carbon atom of the CNN moiety and N_A_ to the nitrogen atom of the amino group. Signs “+” and “−” designate combination of vibrations occurring in the same phase and in the opposite phase, respectively.

**Table 3:** Experimental (Ar, 15 K) and B3LYP/6-311++G(d,p) computed infrared absorptions for *C*-(3-aminophenyl)-nitrilimine **2m** and approximate vibrational assignments.

Ar Matrix ^a^	Computed ^b^		Vibrational Assignment ^c^
	*ν̃*	*A^th^*	
3487	3489.2/3490.8	19.5/19.4	ν_as_(NH_2_)
n.o.	3395.3/3396.6	29.2/29.8	ν_s_(NH_2_)
n.o.	3240.7/3277.1	5.1/31.5	ν(NH)
2238	2236.1	967.0	ν_as_(CNN), **2mP**
2067	2091.1	481.6	ν_as_(CNN), **2mA**
1618	1630.5/1629.9	171.3/157.1	δ(NH_2_)
1599	1605.4/1602.0	88.6/148.9	ν(CC)_ph_
1586	1579.4/1576.9	36.0/50.5	ν(CC)_ph_
1490	1491.4/1492.4	2.2/18.9	ν(CC)_ph_; δ(CH)
n.o.	1454.1/1447.7	38.8/27.5	ν(CC)_ph_
1366	1375.4/1382.6	77.5/63.8	ν(NN) − ν(CC_N_)
n.o.	1321.9/1323.9	10.8/4.5	δ(CH); ν(CC)_ph_
1306	1302.0/1297.2	10.4/9.2	ν(CC)_ph_; ν(CN_A_)
1279	1270.4/1273.6	172.4/85.5	ν(CN_A_); δ(NH); δ(CH)
1246/1237	1239.9	234.3	δ(NH), **2mA**
1221	1220.4	229.7	δ(NH), **2mP**

^a^ Experimental wavenumbers are given in cm^−1^. Only the bands in the 3600–3200 cm^−1^ and 2300–1200 cm^−1^ regions are included. Abbreviations: n.o. = not observed. ^b^ Data extracted from the vibrational calculations carried out for the B3LYP/6-311++G(d,p) lowest energy conformers of **2mA** (**2mA-a**) and **2mP** (**2mP-a**); see Table S3 for details. Values before and after the slash refer to **2mA** and **2mP**, respectively. Calculated harmonic wavenumbers were multiplied by 0.950 (above 2000 cm^−1^) or 0.980 (below 2000 cm^−1^). Vibrations predicted with very low intensities and lacking counterparts in the experimental spectrum were omitted. Vibrational assignment was based on the results provided by the “vibAnalysis” software, supported by ChemCraft animation of the vibrations. ^c^ Abbreviations: ν, stretching; δ, in-plane bending; ph, phenyl ring; s, symmetric; as, antisymmetric; C_N_ refers to the carbon atom of the CNN moiety and N_A_ to the nitrogen atom of the amino group. Sign “−” designates a combination of vibrations occurring in the opposite phase.

**Table 4:** Geometric parameters and Hirshfeld atomic charges computed at the B3LYP/cc-pVTZ level for *C*-phenyl-nitrilimine and its *ortho*-, *meta*-, and *para*-NH_2_ and NO_2_ phenyl-substituted derivatives ^a^.

Allenic-Type Structures					
	*C*-Phenyl-Nitrilimine	*ortho*-NH_2_	*meta*-NH_2_(2mA)	*meta*-NO_2_	*para*-NH_2_(2p)
	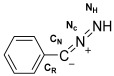	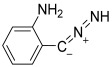	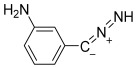	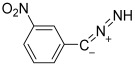	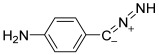
Geometric parameters					
C_R_C_N_ (Å)	1.44	1.44	1.44	1.43	1.43
C_N_N_C_ (Å)	1.18	1.19	1.18	1.18	1.19
N_C_N_H_ (Å)	1.25	1.25	1.25	1.24	1.25
C_R_C_N_N_C_ (º)	142.13	142.33	142.45	144.93	143.04
C_N_N_C_N_H_ (º)	169.82	169.40	169.87	170.40	169.39
C_R_C_N_N_H_H (º)	−99.47	−98.22	−99.46	−104.16	−97.61
Hirshfeld charges					
C_R_	0.002	−0.025	0.001	0.013	−0.019
C_N_	−0.019	−0.023	−0.018	−0.004	−0.025
N_C_	0.049	0.044	0.048	0.058	0.039
N_H_	−0.211	−0.212	−0.215	−0.198	−0.224
**Propargylic-Type Structures**					
	*C*-Phenyl-Nitrilimine	*ortho*-NO_2_	*meta*-NH_2_(2mP)	*meta*-NO_2_	*para*-NO_2_
	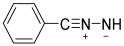	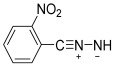	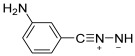	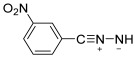	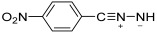
Geometric parameters					
C_R_C_N_ (Å)	1.41	1.40	1.41	1.41	1.41
C_N_N_C_ (Å)	1.17	1.17	1.17	1.17	1.17
N_C_N_H_ (Å)	1.26	1.25	1.26	1.25	1.25
C_R_C_N_N_C_ (º)	179.85	170.92	178.41	179.80	179.92
C_N_N_C_N_H_ (º)	171.76	174.49	171.76	171.70	171.69
C_R_C_N_N_H_H (º)	180.00	180.00	−161.23	180.00	180.00
Hirshfeld charges					
C_R_	0.007	0.016	0.006	0.015	0.020
C_N_	0.043	0.046	0.044	0.044	0.050
N_C_	0.046	0.060	0.044	0.055	0.057
N_H_	−0.239	−0.202	−0.244	−0.219	−0.209

^a^ The data refer to the CNN fragment and were extracted from the optimized geometries of the lowest energy conformers of each molecule (see Tables S3 and S5). Atom labeling is given in the allenic-type structure of *C*-phenyl-nitrilimine.

## Data Availability

Data are contained within the article and the Supplementary Materials.

## Supplementary Materials

The following supporting information can be downloaded at: https://www.mdpi.com/article/10.3390/molecules29153497/s1, Figure S1: B3LYP potential energy scan calculated for the 2*H* tautomeric form of 5-(3-hydroxyphenyl)-tetrazole **1m″**; Figures S2–S3: selected regions of experimental (Ar, 15 K) and theoretical IR spectra of 5-(4-aminoyphenyl)-tetrazole **1p** and 5-(3-aminoyphenyl)-tetrazole **1m**. Figure S4: experimental difference IR spectra in the 3600–3200 cm^−1^ region showing spectral changes after the irradiations of matrix-isolated **1p** at λ = 230 nm followed by irradiations at λ = 380 nm and their comparison with the theoretical IR spectrum of **2p**; Figure S5: fragments of the experimental IR spectra recorded after different times of exposition of matrix-isolated **1p** and **1m** (Ar, 15 K) to UV light at λ = 230 nm; Figure S6: TD-DFT UV spectra computed for the allenic and propargylic bond-shift isomers of **2m**; Figure S7: relaxed potential energy scans as function of the CCN angle calculated for different ring-substituted *C*-phenyl-nitrilimines; Tables S1–S2: bands observed in the IR spectra of **1p** and **1m** (Ar, 15 K) and their comparison with the vibrational data extracted from B3LYP calculations carried out for tautomers **1p″** and **1m″**, including an approximate assignment of the vibration modes; Table S3: structures and B3LYP relative energies calculated for the isomers of **2p** and **2m**; Table S4: structures and B3LYP relative energies calculated for the conformers of 4-aminophenyl-1*H*-diazirine **3p**, 4-aminophenyl-carbodiimide **4p**, 3-aminophenyl-1*H*-diazirine **3m** and 3-aminophenyl-carbodiimide **4m**; Table S5: structures and B3LYP relative energies calculated for the conformers of the *ortho*-NH_2_, *ortho*-NO_2_, and *meta*-NO_2_-substituted *C*-phenyl-nitrilimines; Table S6, Bands observed in the near-IR spectra recorded after isolation of **1m** in an argon matrix at 15 K and after a series of UV irradiations at 230 and 330 nm, and their approximate assignment. Computational data: Cartesian coordinates and energies an extracted from the full geometry optimizations performed at the B3LYP/6-311++G(d,p) level of theory.

## References

[1] Huisgen R., Seidel M., Sauer J., McFarland J., Wallbillich G. (1959). Communications: The Formation of Nitrile Imines in the Thermal Breakdown of 2,5-Disubstituted Tetrazoles. J. Org. Chem..

[2] Padwa A. (1984). 1,3-Dipolar Cycloaddition Chemistry.

[3] Bertrand G., Wentrup C. (1994). Nitrile Imines: From Matrix Characterization to Stable Compounds. Angew. Chem. Int. Ed. Engl..

[4] Huisgen R. (1963). 1,3-Dipolar Cycloadditions. Past and Future. Angew. Chem. Int. Ed. Engl..

[5] Huisgen R. (1963). Kinetics and Mechanism of 1,3-Dipolar Cycloadditions. Angew. Chem. Int. Ed. Engl..

[6] Jamieson C., Livingstone K. (2020). The Nitrile Imine 1,3-Dipole: Properties, Reactivity and Applications.

[7] Molteni G., Ponti A. (2017). The Nitrilimine–Alkene Cycloaddition Regioselectivity Rationalized by Density Functional Theory Reactivity Indices. Molecules.

[8] Stille J.K., Harris F.W. (1968). Polymers from 1,3-dipole addition reactions: The nitrilimine dipole from acid hydrazide chlorides. J. Polym. Sci. Part A Polym. Chem..

[9] Shawali A.S. (1993). Reactions of heterocyclic compounds with nitrilimines and their precursors. Chem. Rev..

[10] An P., Lewandowski T.M., Erbay T.G., Liu P., Lin Q. (2018). Sterically Shielded, Stabilized Nitrile Imine for Rapid Bioorthogonal Protein Labeling in Live Cells. J. Am. Chem. Soc..

[11] Frija L.M.T., Cristiano M.L.S., Gómez-Zavaglia A., Reva I., Fausto R. (2014). Genesis of rare molecules using light-induced reactions of matrix-isolated tetrazoles. J. Photochem. Photobiol., C.

[12] Lucero P.L., Peláez W.J., Riedl Z., Hajós G., Moyano E.L., Yranzo G.I. (2012). Flash vacuum pyrolysis of azolylacroleins and azolylbutadienes. Tetrahedron.

[13] Pagacz-Kostrzewa M., Mucha M., Weselski M., Wierzejewska M. (2013). Conformational properties and photochemistry of new allyl tetrazoles: Matrix isolation FTIR and computational approach. J. Photochem. Photobiol. A.

[14] Wentrup C. (2017). Flash Vacuum Pyrolysis of Azides, Triazoles, and Tetrazoles. Chem. Rev..

[15] Baskir E.G., Platonov D.N., Tomilov Y.V., Nefedov O.M. (2014). Infrared-spectroscopic study of amino-substituted nitrilimines and their photochemical transformations in an argon matrix. Mendeleev Commun..

[16] Bégué D., Qiao G.G., Wentrup C. (2012). Nitrile Imines: Matrix Isolation, IR Spectra, Structures, and Rearrangement to Carbodiimides. J. Am. Chem. Soc..

[17] Ferreira G.A., Nunes C.M., Lopes Jesus A.J., Fausto R. (2023). The meta and para OH Substitution Effect on C-Phenyl-Nitrilimine Bond-Shift Isomers. Eur. J. Org. Chem..

[18] Nunes C.M., Reva I., Fausto R., Bégué D., Wentrup C. (2015). Bond-shift isomers: The co-existence of allenic and propargylic phenylnitrile imines. Chem. Commun..

[19] Nunes C.M., Reva I., Rosado M.T.S., Fausto R. (2015). The Quest for Carbenic Nitrile Imines: Experimental and Computational Characterization of C-Amino Nitrile Imine. Eur. J. Org. Chem..

[20] Nunes C.M., Araujo-Andrade C., Fausto R., Reva I. (2014). Generation and Characterization of a 4π-Electron Three-Membered Ring 1H-Diazirine: An Elusive Intermediate in Nitrile Imine–Carbodiimide Isomerization. J. Org. Chem..

[21] Bégué D., Wentrup C. (2014). Carbenic Nitrile Imines: Properties and Reactivity. J. Org. Chem..

[22] Muchall H.M. (2011). Computational Insight into the Carbenic Character of Nitrilimines from a Reactivity Perspective. J. Phys. Chem. A.

[23] Herges R. (1994). Organizing Principle of Complex Reactions and Theory of Coarctate Transition States. Angew. Chem. Int. Ed. Engl..

[24] Maltsev A., Bally T., Tsao M.-L., Platz M.S., Kuhn A., Vosswinkel M., Wentrup C. (2004). The Rearrangements of Naphthylnitrenes:  UV/Vis and IR Spectra of Azirines, Cyclic Ketenimines, and Cyclic Nitrile Ylides. J. Am. Chem. Soc..

[25] Zhao X., Liu Q., Feng R., Zeng X., Wentrup C. (2019). Photolysis and Pyrolysis of Phenyltetrazoles: Formation of Phenylcarbodiimide, N-Phenylnitrile Imine, Phenylnitrene, Indazole, and Fulvenallene. Eur. J. Org. Chem..

[26] Bugalho S.C.S., Maçôas E.M.S., Cristiano M.L.S., Fausto R. (2001). Low temperature matrix-isolation and solid state vibrational spectra of tetrazole. Phys. Chem. Chem. Phys..

[27] Lopes Jesus A.J., Rosado M.T.S., Reva I., Fausto R., Eusébio M.E., Redinha J.S. (2006). Conformational Study of Monomeric 2,3-Butanediols by Matrix-Isolation Infrared Spectroscopy and DFT Calculations. J. Phys. Chem. A.

[28] Rosado M.T.S., Lopes Jesus A.J., Reva I.D., Fausto R., Redinha J.S. (2009). Conformational Cooling Dynamics in Matrix-Isolated 1,3-Butanediol. J. Phys. Chem. A.

[29] Barnes A.J. (1984). Matrix isolation vibrational spectroscopy as a tool for studying conformational isomerism. J. Mol. Struct..

[30] Jesus A.J.L., Rosado M.T.S., Reva I., Fausto R., Eusébio M.E.S., Redinha J.S. (2008). Structure of Isolated 1,4-Butanediol: Combination of MP2 Calculations, NBO Analysis, and Matrix-Isolation Infrared Spectroscopy. J. Phys. Chem. A.

[31] Elpern B., Nachod F.C. (1950). Absorption Spectra and Structure of Some Tetrazoles. J. Am. Chem. Soc..

[32] Maier G., Eckwert J., Bothur A., Reisenauer H.P., Schmidt C. (1996). Photochemical Fragmentation of Unsubstituted Tetrazole, 1,2,3-Triazole, and 1,2,4-Triazole: First Matrix-Spectroscopic Identification of Nitrilimine HCNNH. Liebigs Annalen.

[33] Bégué D., Dargelos A., Wentrup C. (2020). Aryl Nitrile Imines and Diazo Compounds. Formation of Indazole, Pyridine N-Imine, and 2-Pyridyldiazomethane from Tetrazoles. J. Org. Chem..

[34] Bégué D., Santos-Silva H., Dargelos A., Wentrup C. (2017). Imidoylnitrenes R′C(═NR)–N, Nitrile Imines, 1H-Diazirines, and Carbodiimides: Interconversions and Rearrangements, Structures, and Energies at DFT and CASPT2 Levels of Theory. J. Phys. Chem. A.

[35] Ismael A., Fausto R., Cristiano M.L.S. (2016). Photochemistry of 1- and 2-Methyl-5-aminotetrazoles: Structural Effects on Reaction Pathways. J. Org. Chem..

[36] Lopes Jesus A.J., Nunes C.M., Fausto R., Reva I. (2018). Conformational control over an aldehyde fragment by selective vibrational excitation of interchangeable remote antennas. Chem. Commun..

[37] Nunes C.M., Pereira N.A.M., Viegas L.P., Pinho e Melo T.M.V.D., Fausto R. (2021). Inducing molecular reactions by selective vibrational excitation of a remote antenna with near-infrared light. Chem. Commun..

[38] Lopes Jesus A.J., Nunes C.M., Reva I., Pinto S.M.V., Fausto R. (2019). Effects of Entangled IR Radiation and Tunneling on the Conformational Interconversion of 2-Cyanophenol. J. Phys. Chem. A.

[39] Yoshida K.-i., Iiba E., Nozaki Y., Hirai K., Takahashi Y., Tomioka H., Lin C.-T., Gaspar P.P. (2004). Di(9-anthryl)carbene Revisited. Product Analysis and Spectroscopic Studies. Bull. Chem. Soc. Jpn..

[40] DePinto J.T., McMahon R.J. (1993). Structure and rearrangements of 1,3-diphenylpropynylidene. J. Am. Chem. Soc..

[41] Carra C., Nussbaum R., Bally T. (2006). Experimental and Theoretical Study of 2,6-Difluorophenylnitrene, Its Radical Cation, and Their Rearrangement Products in Argon Matrices. ChemPhysChem.

[42] Reva I.D., Stepanian S.G., Adamowicz L., Fausto R. (2003). Missing conformers. Comparative study of conformational cooling in cyanoacetic acid and methyl cyanoacetate isolated in low temperature inert gas matrixes. Chem. Phys. Lett..

[43] Sponsler M.B., Jain R., Coms F.D., Dougherty D.A. (1989). Matrix-isolation decay kinetics of triplet cyclobutanediyls. Observation of both Arrhenius behavior and heavy-atom tunneling in carbon-carbon bond-forming reactions. J. Am. Chem. Soc..

[44] Hirshfeld F.L. (1977). Bonded-atom fragments for describing molecular charge densities. Theor. Chim. Acc..

[45] Wiberg K.B., Rablen P.R. (2018). Atomic Charges. J. Org. Chem..

[46] Liu S. (2014). Where does the electron go? The nature of ortho/para and meta group directing in electrophilic aromatic substitution. J. Chem. Phys..

[47] Liu S. (2015). Quantifying Reactivity for Electrophilic Aromatic Substitution Reactions with Hirshfeld Charge. J. Phys. Chem. A.

[48] Nikolova V., Cheshmedzhieva D., Ilieva S., Galabov B. (2019). Atomic Charges in Describing Properties of Aromatic Molecules. J. Org. Chem..

[49] Frisch M.E., Trucks G.W., Schlegel H.B., Scuseria G.E., Robb M., Cheeseman J.R., Scalmani G., Barone V., Petersson G.A., Nakatsuji H. (2016). GAUSSIAN 16.

[50] Becke A.D. (1993). Density-functional thermochemistry. III. The role of exact exchange. J. Chem. Phys..

[51] Lee C.T., Yang W.T., Parr R.G. (1988). Development of the Colle-Salvetti correlation-energy formula into a functional of the electron-density. Phys. Rev. B.

[52] Vosko S.H., Wilk L., Nusair M. (1980). Accurate spin-dependent electron liquid correlation energies for local spin density calculations: A critical analysis. Can. J. Phys..

[53] Teixeira F., Cordeiro M.N.D.S. (2019). Improving Vibrational Mode Interpretation Using Bayesian Regression. J. Chem. Theory Comput..

[54] Zhurko G.A.C. (2016). Chemcraft, Version 1.8. http://www.chemcraftprog.com.

[55] Barone V. (2004). Anharmonic vibrational properties by a fully automated second-order perturbative approach. J. Chem. Phys..

[56] Bloino J., Barone V. (2012). A second-order perturbation theory route to vibrational averages and transition properties of molecules: General formulation and application to infrared and vibrational circular dichroism spectroscopies. J. Chem. Phys..

[57] Bauernschmitt R., Ahlrichs R. (1996). Treatment of electronic excitations within the adiabatic approximation of time dependent density functional theory. Chem. Phys. Lett..

[58] Stratmann R.E., Scuseria G.E., Frisch M.J. (1998). An efficient implementation of time-dependent density-functional theory for the calculation of excitation energies of large molecules. J. Chem. Phys..

[59] Adamo C., Barone V. (1999). Toward reliable density functional methods without adjustable parameters: The PBE0 model. J. Chem. Phys..

[60] Zhao Y., Truhlar D.G. (2005). Design of Density Functionals That Are Broadly Accurate for Thermochemistry, Thermochemical Kinetics, and Nonbonded Interactions. J. Phys. Chem. A.

