# Supplementary material for: Photochemical Generation and Characterization of C-Aminophenyl-Nitrilimines: Insights on Their Bond-Shift Isomers by Matrix-Isolation IR Spectroscopy and Density Functional Theory Calculations
Source: Molecules. 2024 Jul 25;29(15):3497. doi: 10.3390/molecules29153497 (PMC11314218; doi:10.3390/molecules29153497)
Supplement: Supplementary file 1 [file molecules-29-03497-s001.zip › molecules-3127007-supplementary.pdf]

Supplementary Material

Photochemical Generation and Characterization of *C*-Aminophenyl-Nitrilimines: Insights on Their Bond-Shift Isomers by Matrix-Isolation IR Spectroscopy and Density Functional Theory Calculations

A. J. Lopes Jesus,[1,]* Cláudio M. Nunes,[2] Gil A. Ferreira,[2] Kiarash Keyvan,[2] R. Fausto[2,3]

[1] University of Coimbra, CQC-IMS, Faculty of Pharmacy, 3004-295, Coimbra, Portugal.

[2] University of Coimbra, CQC-IMS, Department of Chemistry, 3004-535, Coimbra, Portugal.

[3] Istanbul Kultur University, Faculty Sciences and Letters, Department of Physics, 34158 Bakirkoy, Istanbul, Turkey

* Corresponding author:

E-mail address: ajorge@ff.uc.pt

Table of Contents:

1. FIGURES S2

2. TABLES S9

3. COMPUTATIONAL DATA S15

1. FIGURES

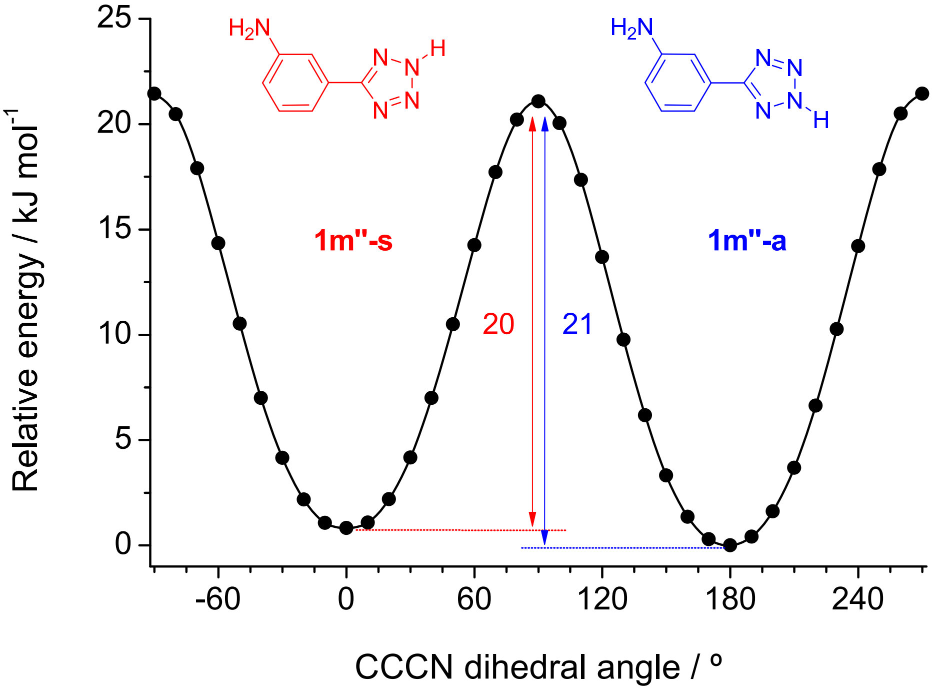

Figure S1. B3LYP/6-311++G(d,p) relaxed potential energy scan calculated for the 2*H*-tautomer of 5-(3-hydroxyphenyl)-tetrazole **1m"** as a function of the internal rotation around the inter-rings C-C bond.

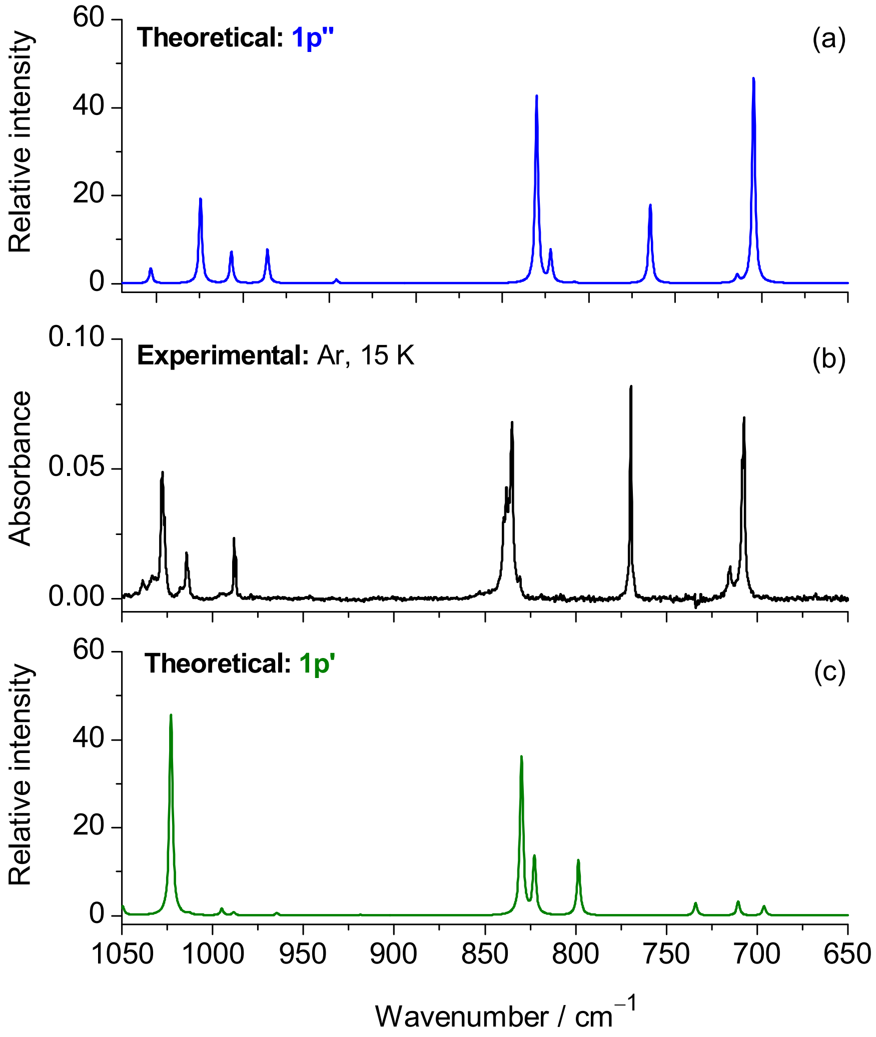

Figure S2. (b) Selected region of the experimental IR spectrum of 5-(4-aminoyphenyl)-tetrazole **1p** isolated in an Ar matrix at 15 K, compared with the B3LYP/6-311++G(d,p) theoretical IR spectra calculated for (c) tautomer **1p'** (green line), and (a) tautomer **1p''** (blue line). Calculated wavenumbers were scaled by 0.98 while calculated intensities were not scaled.

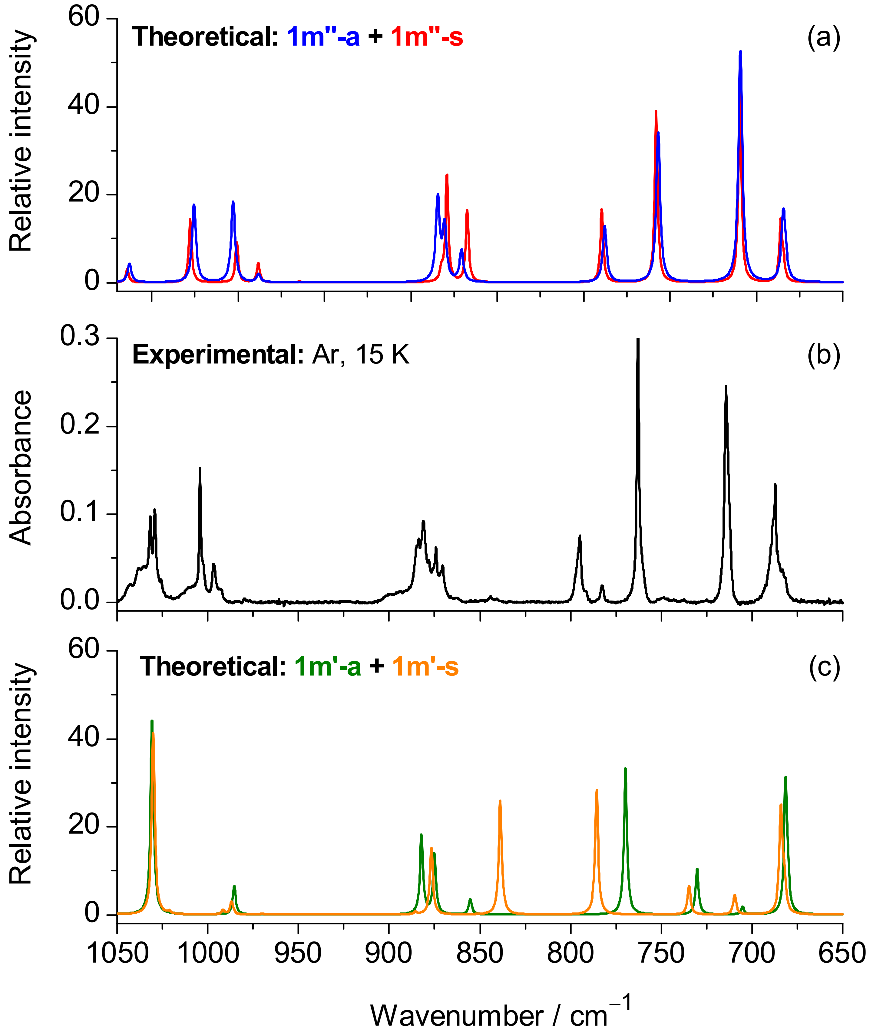

Figure S3. (b) Selected region of the experimental IR spectrum of 5-(3-aminoyphenyl)-tetrazole **1m** isolated in an Ar matrix at 15 K, compared with the B3LYP/6-311++G(d,p) theoretical IR spectra calculated for (c) the two conformers of tautomer **1m'** (**1m'-a**, green line and **1m'-s**, orange line), and (a) the two conformers of tautomer **1m''** (**1m''-a**, blue line and **1m''-s**, red line). Calculated wavenumbers were scaled by 0.98 while calculated intensities were not scaled.

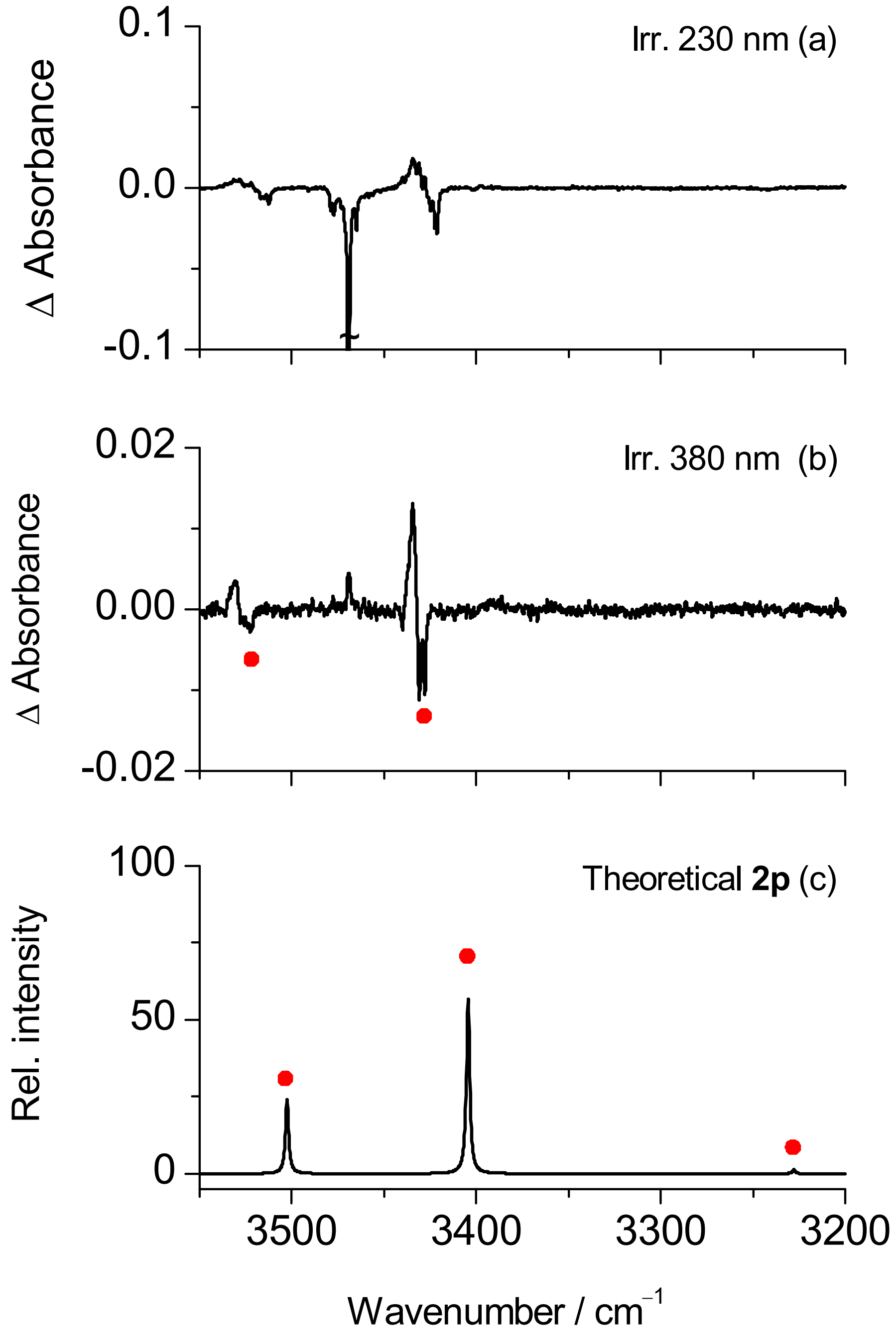

Figure S4. Experimental difference IR spectra (3600–3200 cm^{-1}) showing changes (a) after irradiation of matrix isolated **1p** (Ar, 15 K) at λ = 230 nm for ~1 min and (b) after subsequent ~26 min of irradiation at λ = 380 nm (the negative bands labeled by red circles are due to the consumed photoproduct assigned to **2p**); (c) Theoretical IR spectrum of the most stable conformer of *C*-(4-aminophephenyl)-nitrilimine **2p** calculated at the B3LYP/6-311++G(d,p) level (see Table S3 for details).

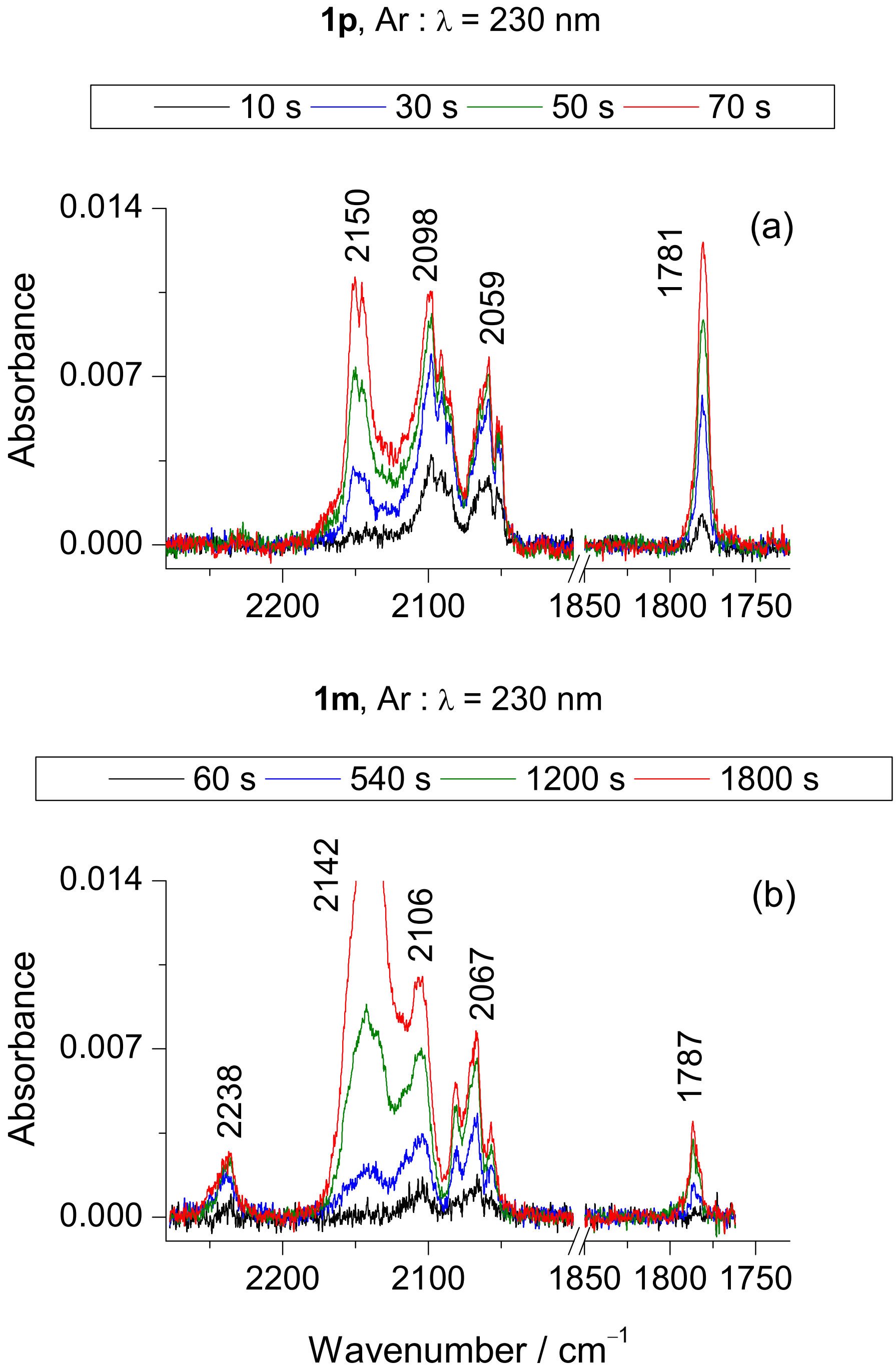

Figure S5. Fragments of experimental IR spectra recorded after different times of exposition of (a) matrix isolated 5-(4-aminoyphenyl)-tetrazole **1p** and (b) 5-(3-aminoyphenyl)-tetrazole **1m** to UV light at λ = 230 nm, showing the kinetic grow of the most characteristic bands of the photoproducts.

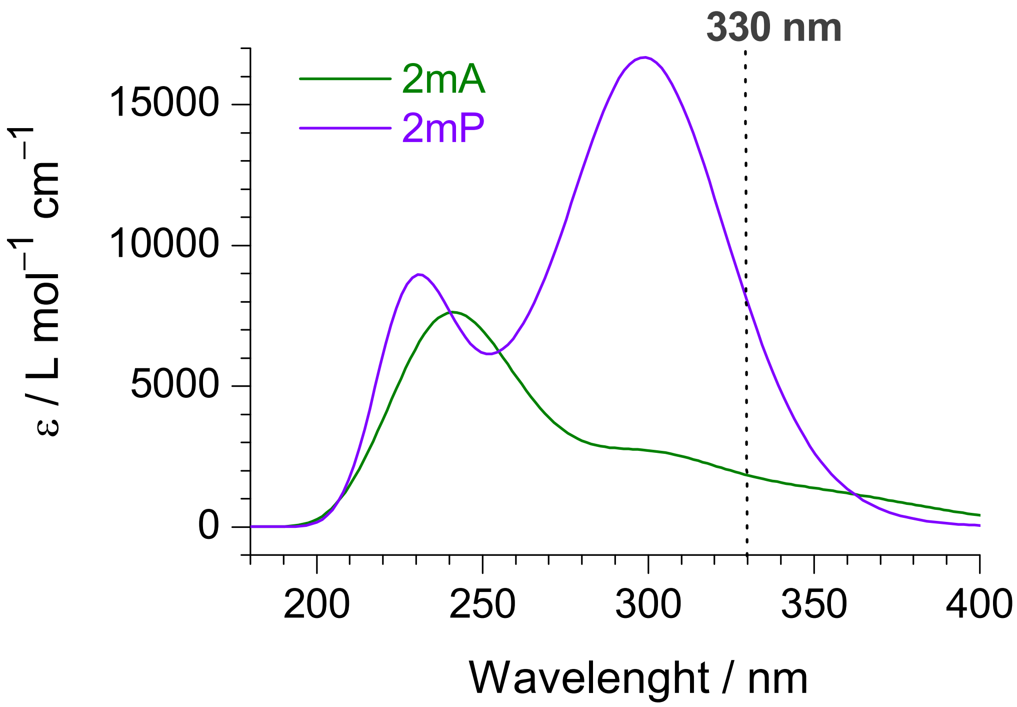

Figure S6. Theoretical UV spectra of the allenic (**2mA**) and propargylic (**2mP**) bond shift isomers of *C*-(3-aminophephenyl)-nitrilimine **2m** obtained from TD-DFT calculations carried out at the B3LYP/6-311++G(d,p) level. Vertical dotted line at 330 nm represents the position of narrowband UV-irradiations performed in this study to induce the consumption of **2m**.

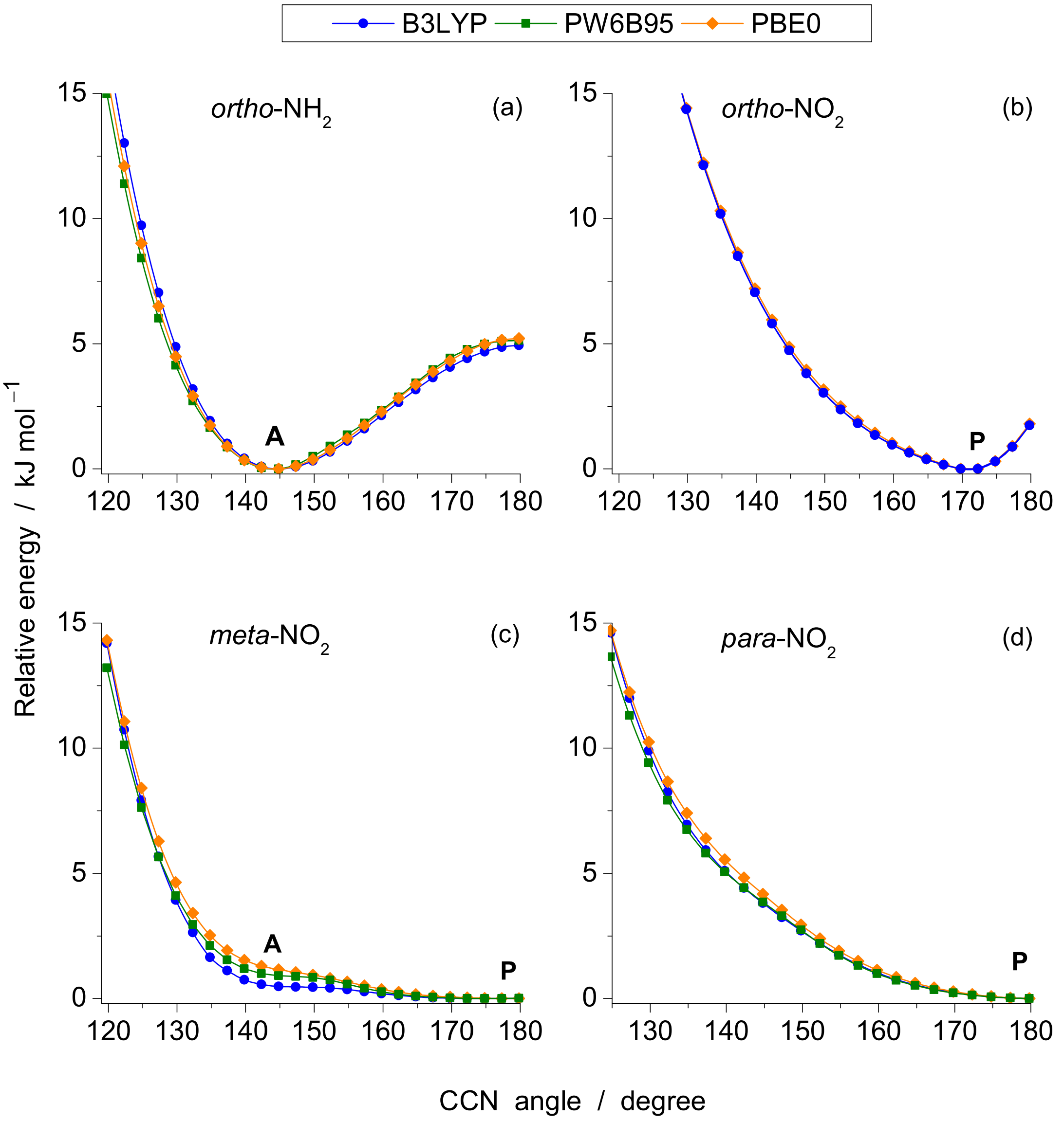

Figure S7. Relaxed potential energy scans as function of the CCN angle calculated for different ring substituted *C*-phenyl-nitrilimines: (a) *ortho*-NH_2; (b) *ortho*-NO_2; (c) *meta*-NO_2; (d) *para*-NO_2. The scans were computed by combining the B3LYP (blue), PW6B95 (green), and PBE0 (orange) DFT functionals and the 6-311++G(d,p) basis set. Along the scans, the value of the CCN angle was incrementally fixed in steps of 2.5 degrees, and all other geometrical parameters were fully optimized.

2. TABLES

Table S1. Bands observed in the IR spectrum of 5-(4-aminophenyl)-tetrazole **1p** isolated in an Ar matrix at 15 K, compared with the harmonic wavenumbers ($\tilde{\nu}$ /cm^{-1}) and absolute intensities (A^{th} / km mol^{-1}) computed for tautomer **1p''** of this molecule at the B3LYP/6-311++G(d,p) level, including an approximate assignment of the vibration modes.

Experimental[a]		Calc. (**1p''**)[b]		Approximate assignment[c]
$\tilde{\nu}$	*I*	$\tilde{\nu}$	A^{th}	
3513	w	3494.6	20.5	$\nu_{as}(NH_2)$
3469 (split)	s	3457.4	152.7	ν(NH)
3421	w	3398.6	41.8	$\nu_s(NH_2)$
1625	s	1632.4	271.4	$\delta(NH_2)$
1608	w	1618.6	32.1	$\nu(CC)_{ph}$
1573	w	1580.9	4.7	$\nu(CC)_{ph}$
1555	w	1545.2	0.6	$\nu(CC)_{ph\text{-}tz}$
1498 / 1483	s, split	1485.2	117.5	δ(CNN); δ(NCN); δ(NH); $\nu(CN)_{tz}$
1449	s, split	1446.3	157.5	$\nu(CC)_{ph\text{-}tz}$; δ(NCN)
1428	w	1435.4	5.7	$\nu(CC)_{ph}$; δ(NH)
1318	w	1328.5	24.3	ν(NN); $\nu(CN)_{tz}$; $\nu(CC)_{ph}$; δ(CH)
1294	s, split	1284.4	101.4	$\nu(CC_{NH_2})$
1186	m, split	1181.2	43.9	$\nu(CC)_{ph}$; δ(CH)
1180	m	1172.7	26.4	ν(NN); δ(CNN)
1133	w, split	1129.5	9.1	$\nu(CC)_{ph}$; δ(CH)
1111	w	1105.7	2.7	ν(NN); ν(NN); δ(NNN)
1103	w, split	1092.3	6.5	ν(NN)
1056	w	1053.3	3.4	$\rho(NH_2)$
1028	m	1024.5	19.4	δ(NNN)
1014	w	1006.7	7.1	$\delta(CCC)_{ph}$
988	w, split	985.8	7.8	δ(CNN)
835	m, split	830.0	42.7	$\gamma(C)_{ph}$
830	w, sh	822.0	7.1	$\nu(CC)_{ph}$; $\delta(CCC)_{ph}$
770	m	764.4	17.9	$\gamma(C)_{tz}$
715	m	714.0	1.7	$\gamma(C)_{ph}$
707	m	704.6	46.8	$\gamma(N)_{tz}$
631	w, split	626.8	5.2	$\delta(CCC)_{ph}$

[a] Experimental wavenumbers are given in cm^{-1}. Bands' intensities are expressed in qualitative terms: s = strong; m = medium; w = weak; sh = shoulder. Bands falling in the 3100−2800 cm^{-1} region (assigned to the CH stretching vibrations), as well as those below 600 cm^{-1} and other weak bands with no correspondence in the theoretical spectrum (most likely due to the presence of trace amounts of tautomer **1p'**), were not investigated. [b] Calculated harmonic wavenumbers were multiplied by 0.950 (above 2000 cm^{-1}) or 0.980 (below 2000 cm^{-1}). Vibrations predicted with very low predicted intensities and lacking counterparts in the experimental spectrum were omitted. [c]Approximate assignment was based on the results provided by the "vibAnalysis" software, supported by ChemCraft animation of the vibrations. Abbreviations: ν, stretching; δ, in-plane bending; γ, out-of-plane bending; ρ, rocking; tz, tetrazole ring; ph, phenyl ring.

Table S2. Bands observed in the IR spectrum of 5-(3-aminophenyl)-tetrazole **1m** isolated in an Ar matrix at 15 K, compared with the harmonic wavenumbers ($\tilde{\nu}$ /cm^{-1}) and absolute intensities (A^{th} / km mol^{-1}) computed for the **1m''-a** and **1m''-s** isomers of this molecule at the B3LYP/6-311++G(d,p) level, including an approximate assignment of the vibration modes.

Experimental[a]		Calc. (**1m''-a**)[b]		Calc. (**1m''-s**)[b]		Approximate assignment[c]
$\tilde{\nu}$	*I*	$\tilde{\nu}$	A^{th}	$\tilde{\nu}$	A^{th}	
3507	w	3487.7	10.5	3486.3	6.9	$\nu_{as}(NH_2)$
3467	s, split	3454.5	86.2	3455.0	57.9	$\nu(NH)$
3417	w	3393.9	14.3	3393.0	9.0	$\nu_s(NH_2)$
1621	s	1630.3	93.6	1630.2	63.3	$\delta(NH_2)$
1612	w	1614.9	10.2	1615.1	7.6	$\nu(CC)_{ph}$
1600	m	1595.2	17.1	1595.6	11.4	$\nu(CC)_{ph}$
1531	m	1524.3	21.5	1524.8	10.4	$\nu(CC)_{ph\text{-}tz}$; $\nu(CN)_{tz}$
1498	s, split	1494.9	46.2	–	–	$\delta(CNN)$; $\nu(CN)_{tz}$; $\delta(NH)$
1476	w	–	–	1483.4	4.7	$\nu(NN)$; $\nu(CN)_{tz}$; $\delta(NH)$
1469	m	–	–	1468.3	47.8	$\nu(CC)_{ph}$; $\nu(CN)_{tz}$
1445	w	1450.5	13.1	–	–	$\delta(NH)$
1437	m	1434.4	29.9	–	–	$\delta(NCN)$; $\nu(CC)_{ph\text{-}tz}$; $\nu(CN)_{tz}$
1428	w	–	–	1429.1	9.3	$\delta(NCN)$; $\nu(NN)$; $\nu(CC)_{ph\text{-}tz}$
1327	w	1330.1	0.1	1328.2	6.3	$\nu(CC)_{ph}$
1324	w	1326.8	6.8	1326.6	11.0	$\delta(CH)$
1315	w, split	1299.6	15.1	–	–	$\nu(CC_{NH_2})$; $\nu(CN)_{tz}$; $\nu(NN)$
1277	w	–	–	1283.5	8.3	$\nu(NN)$; $\nu(CN)_{tz}$
1272	w, sh	–	–	1270.6	9.9	$\nu(CC_{NH_2})$
1253	m	1256.1	30.9	–	–	$\nu(NN)$; $\nu(CC_{NH_2})$
1225	w	1238.8	7.9	1238.4	3.6	$\nu(NN)$; $\nu(CN)_{tz}$
1195	w	1176.7	7.7	1175.7	12.6	$\nu(NN)$; $\delta(NNN)$
1170	w, split	1168.5	1.3	1170.8	3.3	$\delta(CH)$
1135	w	1119.1	4.0	1119.4	0.5	$\nu(CC)_{ph}$; $\rho(NH_2)$; $\delta(CH)$
1115	w	1094.8	3.2	1094.8	3.0	$\nu(NN)$; $\delta(NNN)$
1089	w	1084.9	2.1	1084.4	2.0	$\nu(NN)$
1072	w, split	1062.8	2.4	1064.1	1.2	$\rho(NH_2)$
1030	m, split	1025.5	10.1	1027.7	5.6	$\delta(NNN)$
1004	m	1002.8	10.5	1000.7	3.6	$\delta(CNN)$; $\nu(CN)$
997	w	988.1	1.0	988.2	1.7	$\delta(CCC)_{ph}$
884	m	884.4	10.7	–	–	$\nu(CC)_{ph}$; $\delta(CCC)_{ph}$
881	m	880.5	7.0	879.1	9.5	$\nu(CC)_{ph}$; $\nu(CN)_{ph}$; $\delta(CCC)_{ph}$
874	w, split	870.6	4.1	867.3	6.4	$\gamma(C)_{ph}$; $\gamma(CH)$
795	w	787.7	7.3	789.4	6.5	$\gamma(C)_{ph}$; $\gamma(C)_{tz}$; $\gamma(CH)$
763	s	756.8	19.5	758.0	15.2	$\gamma(C)_{tz}$
714	s	709.2	30.0	709.5	17.3	$\gamma(N)_{tz}$
687	m	684.5	8.0	685.9	5.3	$\gamma(C)_{ph}$
683	w, sh	683.3	2.7	684.2	1.4	$\delta(CCC)_{ph}$

[a] Experimental wavenumbers are given in cm^{-1}. Bands' intensities are expressed in qualitative terms: s = strong; m = medium; w = weak; sh = shoulder. Bands falling in the 3100−2800 cm^{-1} region (assigned to the CH stretching vibrations), as well as those below 600 cm^{-1} and other weak bands with no correspondence in the theoretical spectrum (most likely due to the presence of trace amounts of tautomer **1m'**), were not investigated.[b] Calculated harmonic wavenumbers were multiplied by 0.950 (above 2000 cm^{-1}) or 0.980 (below 2000 cm^{-1}) and the intensities were scaled by the predicted gas-phase populations for the two **1m''** conformers (57.1% for **1m''-a** and 39.0% for **1m''-s**, see Table 1). Vibrations predicted with very low predicted intensities and lacking counterparts in the experimental spectrum were omitted. [c]Approximate assignment was based on the results provided by the "vibAnalysis" software, supported by ChemCraft animation of the vibrations. Abbreviations: ν, stretching; δ, in-plane bending; γ, out-of-plane bending; ρ, rocking; tz, tetrazole ring; ph, phenyl ring.

Table S3. Structures and relative zero-point corrected energies (in kJ mol^{-1}) computed at the B3LYP/6-311++G(d,p) level of theory for the conformers of *C*-(4-aminophenyl)-nitrilimine **2p** and *C*-(3-aminophenyl)-nitrilimine **2m**.[a]

C-(4-aminophenyl)-nitrilimine **2p**

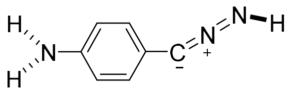	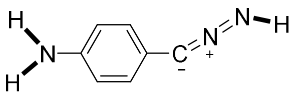
**2p-a**	**2p-s**
0.00	0.10

C-(3-aminophenyl)-nitrilimine **2m**

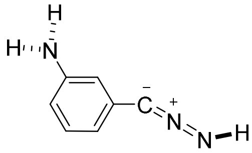	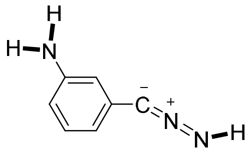	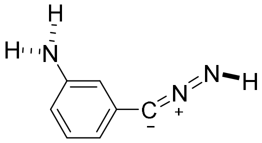	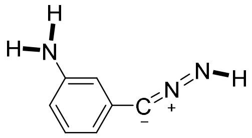
**2mA-aa**	**2mA-as**	**2mA-sa**	**2mA-ss**
1.38	1.57	1.53	1.60
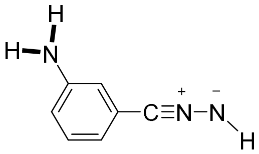	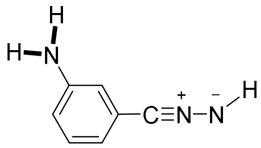		
**2mP-a**	**2mP-s**		
0.00	0.44		

[a] In the isomer designations, **A** and **P** indicate allenic and propargylic nitrilimines, respectively, while the one or two small letters after the hyphen are used to identify the relative orientation of specific groups or atoms (**s** = *syn*; **a** = *anti*). For **2p**, the letter indicates whether the NH and NH_2 hydrogen atoms are pointing to the same side or to opposite sides of the ring plane. For **2mA**, the first letter indicates the orientation of the CNN fragment with respect to the NH_2 group, while the second letter indicates whether the NH and NH_2 hydrogen atoms are pointing to the same side or to opposite sides of the ring plane. Finally, for **2mP**, the letter indicates the orientation of the NH relative to the NH_2 group.

Table S4. Structures and relative zero-point corrected energies (in kJ mol^{-1}) computed at the B3LYP/6-311++G(d,p) level of theory for the conformers of 4-aminophenyl-1*H*-diazirine **3p**, 4-aminophenyl-carbodiimide **4p**, 3-aminophenyl-1*H*-diazirine **3m**, 4-aminophenyl-carbodiimide **4m**. [a]

4-aminophenyl-1*H*-diazirine **3p**	
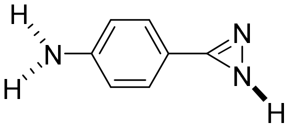	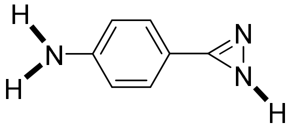
**3p-a**	**3p-s**
0.00	0.12
4-aminophenyl-carbodiimide **4p**	
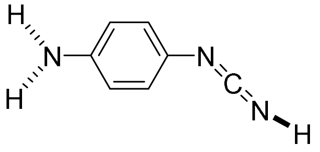	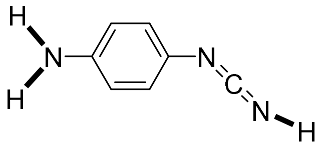
**4p-a**	**4p-s**
0.00	0.15
3-aminophenyl-1*H*-diazirine **3m**	
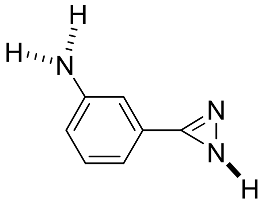	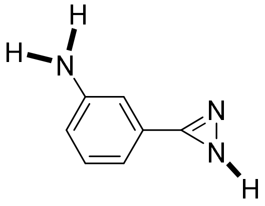
**3m-a**	**3m-s**
0.00	0.21
3-aminophenyl-carbodiimide **4p**	
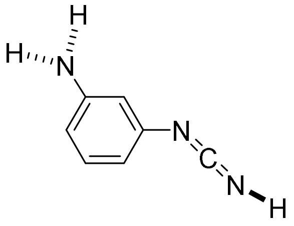	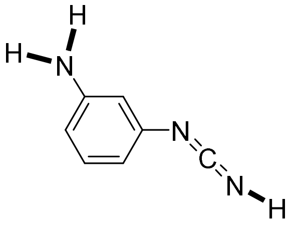
**4p-a**	**4p-s**
0.00	0.18

[a] The small letter after the hyphen indicates whether the NH and NH_2 hydrogen atoms are pointing to the same side (**s** = *syn*) or to opposite sides (**a** = *anti*) of the ring plane.

Table S5. Structures and relative zero-point corrected energies (in kJ mol^{-1}) computed at the B3LYP/cc-pVTZ level of theory for the conformers of the *ortho*-NH_2, *ortho*-NO_2 and *meta*-NO_2 substituted *C*-phenyl-nitrilimines.

***ortho*-NH_2**	
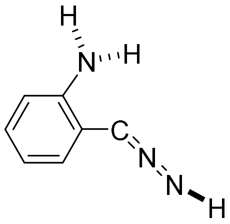	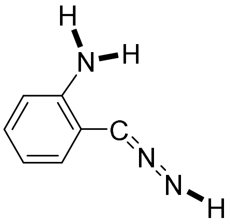
**A-a**	**A-s**
0.00	0.40
***ortho*-NO_2**	
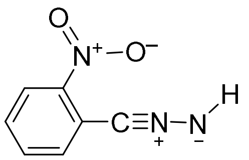	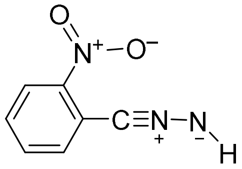
**P-a**	**P-s**
4.04	0.00
***meta*-NO_2**	
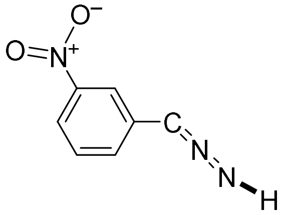	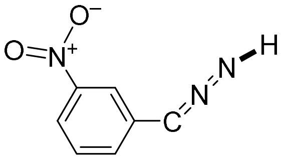
**A-a**	**A-s**
1.47	2.25
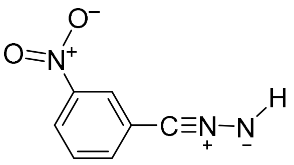	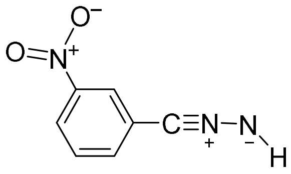
**P-a**	**P-s**
0.88	0.00

[a] The isomers are identified by capital letters "**A**" or "**P**" denoting allenic or propargylic structures, respectively, followed by a small letter **s** (*syn*) or **a** (*anti*). For *ortho*-NH_2, the letter indicates whether the NH and NH_2 hydrogen atoms are pointing to the same side or to opposite sides of the ring plane. For *ortho*-NO_2 and the Propargylic form of *meta*-NO_2, the letter indicates the orientation of the NH relative to the NO_2 group. Finally, for the allenic form of *meta*-NO_2, the letter refers to the orientation of the CNN fragment with respect to the NO_2 group.

Table S6. Bands observed in the near-IR spectra recorded after isolation of **1m** in an argon matrix at 15 K and after a series of UV irradiations at λ = 230 nm, followed by irradiations at λ = 330 nm, and their approximate assignment.

Experimental [a]	Calc.[b] $\tilde{\nu}$	Calc.[b] A^{th}	Approximate assignment
6936	6948.3	2.1	$2\nu_{as}(NH_2)$, **1m''-a**
	6923.7	2.0	$2\nu_{as}(NH_2)$, **1m''-s**
n.o.	6942.6	2.0	$2\nu_{as}(NH_2)$, **3m**
	6931.0	2.1	$2\nu_{as}(NH_2)$, **2mA**
	6925.6	2.1	$2\nu_{as}(NH_2)$, **4m**
6798, **6787**, 6796, 6730	6839.2	2.9	$2\nu(NH)$, **1m''-a**
	6805.3	1.3	$\nu_{as}(NH_2) + \nu_s(NH_2)$, **1m''-a**
	6796.0	6.6	$2\nu(NH)$, **1m''-s**
	6791.2	1.9	$2\nu_s(NH_2)$, **1m''-a**
	6783.1	1.4	$\nu_{as}(NH_2) + \nu_s(NH_2)$, **1m''-s**
	6772.3	2.0	$2\nu_s(NH_2)$, **1m''-s**
6750 (broad)	6800.8	1.3	$\nu_{as}(NH_2) + \nu_s(NH_2)$, **3m**
	6788.2	1.9	$2\nu_s(NH_2)$, **3m**
	6789.8	1.4	$\nu_{as}(NH_2) + \nu_s(NH_2)$, **2mA**
	6778.4	2.1	$2\nu_s(NH_2)$, **2mA**
	6784.4	1.4	$\nu_{as}(NH_2) + \nu_s(NH_2)$, **4m**
	6773.2	2.1	$2\nu_s(NH_2)$, **4m**
6643	6659.6	12.8	$2\nu(NH)$, **4m**
6307	6287.5	14.9	$2\nu(NH)$, **2mA**
n.o.	6204.2	6.3	$2\nu(NH)$, **3m**

[a] Experimental wavenumbers ($\tilde{\nu}$) are given in cm^{-1}. n.o. = not observed. [b] Obtained from B3LYP/6-311++G(d,p) anharmonic vibrational calculations (wavenumbers were not scaled). Note that overtones and combination modes corresponding to **2mP** were not included as this species is selectively consumed in the first minutes of irradiation at λ = 330 nm.

3. COMPUTATIONAL DATA

Cartesian coordinates extracted from full geometry optimizations performed at the B3LYP/6-311++G(d,p) level of theory for the experimentally studied molecules

5-(4-aminophenyl)-tetrazole **1p**

	**1p''**				**1p'**		
N	-2.256884	1.103723	-0.001253	N	-2.335376	1.014706	-0.110259
N	-3.474301	0.573763	0.003318	N	-3.615731	0.574732	-0.063172
N	-3.500882	-0.750136	0.006949	N	-3.543783	-0.702291	0.076721
N	-2.253445	-1.127079	0.005012	N	-2.255332	-1.122989	0.125217
C	-1.492934	0.009669	-0.000075	C	-1.497324	-0.042289	0.007548
C	-0.032181	0.012359	-0.003546	C	-0.040382	-0.002580	0.002326
C	0.690252	1.213435	-0.002871	C	0.679723	1.198145	0.065747
C	2.076261	1.210363	-0.004030	C	2.066140	1.204430	0.059520
C	2.790274	0.000237	-0.004376	C	2.785319	-0.000686	-0.006096
C	2.066126	-1.202970	-0.007063	C	2.063988	-1.206479	-0.065546
C	0.679691	-1.194813	-0.005928	C	0.680282	-1.205496	-0.059707
N	4.179657	-0.005007	-0.057675	N	4.169651	0.000827	-0.062726
H	4.646705	0.833404	0.252612	H	4.644964	0.827391	0.264843
H	4.639581	-0.849783	0.245994	H	4.640920	-0.858337	0.174577
H	-4.316369	1.131634	0.002963	H	-2.147546	1.996324	-0.239776
H	0.156054	2.155989	-0.000530	H	0.163557	2.150183	0.134111
H	2.617148	2.151192	-0.008542	H	2.600780	2.147101	0.107701
H	2.598694	-2.148493	-0.013730	H	2.600214	-2.148077	-0.120840
H	0.134236	-2.130471	-0.005876	H	0.134622	-2.139745	-0.103838

C-(4-aminophenyl)-nitrilimine **2p**

	**2p-a**				**2p-s**		
C	0.553933	0.304378	-0.007460	C	0.553947	0.304180	-0.011279
C	-0.348352	1.380973	-0.002586	C	-0.348243	1.380879	-0.005486
C	-1.717139	1.168843	0.008901	C	-1.717059	1.168957	0.002893
C	-2.238460	-0.136297	0.008750	C	-2.238604	-0.136156	0.000457
C	-1.339830	-1.219346	0.001480	C	-1.340075	-1.219298	-0.011749
C	0.026642	-1.002260	-0.005547	C	0.026432	-1.002370	-0.015364
C	1.968675	0.539999	-0.027025	C	1.968766	0.539674	-0.022943
N	3.037758	0.028340	0.031075	N	3.037698	0.028114	0.038523
N	4.201142	-0.416324	-0.128347	N	4.202042	-0.415526	-0.117002
N	-3.605420	-0.354031	0.066453	N	-3.605914	-0.353180	-0.043119
H	4.653589	-0.495669	0.786868	H	4.649975	-0.498537	0.800106
H	0.041950	2.391595	-0.009641	H	0.042218	2.391465	-0.007073
H	-2.394883	2.016090	0.018244	H	-2.394748	2.016293	0.004347
H	-4.205829	0.407265	-0.209926	H	-3.939283	-1.262985	0.235020
H	-3.940957	-1.260312	-0.220777	H	-4.204144	0.405373	0.244929
H	-1.724778	-2.234016	0.005573	H	-1.725197	-2.233865	-0.021649
H	0.703732	-1.848601	-0.013688	H	0.703411	-1.848800	-0.023668

4-aminophenyl-1*H*-diazirine **3p**

	3p-a				3p-s		
C	-0.696132	0.070885	-0.006798	C	-0.696205	0.070804	-0.01069
C	0.060149	1.255053	0.014017	C	0.060092	1.255026	0.008075
C	1.440800	1.206191	0.022943	C	1.440738	1.206353	0.014004
C	2.114516	-0.032282	0.007948	C	2.114623	-0.032128	0.000213
C	1.355194	-1.216178	-0.013543	C	1.355281	-1.216085	-0.02274
C	-0.028352	-1.161765	-0.020832	C	-0.028265	-1.161748	-0.02741
C	-2.132431	0.108815	-0.016971	C	-2.132511	0.108693	-0.01312
N	-3.137221	0.851423	-0.046840	N	-3.137467	0.851371	-0.0352
N	-3.213061	-0.803610	-0.072359	N	-3.213593	-0.803537	-0.06364
N	3.493872	-0.079728	0.061272	N	3.494007	-0.078032	-0.04027
H	-3.456007	-1.060106	0.890717	H	-3.450594	-1.061966	0.90042
H	-0.448273	2.212444	0.022137	H	-0.448388	2.212361	0.018898
H	2.015552	2.126117	0.043465	H	2.015452	2.126474	0.024318
H	4.003144	0.753213	-0.188712	H	3.945180	-0.945452	0.203428
H	3.945577	-0.938989	-0.209485	H	4.002455	0.747854	0.233107
H	1.860391	-2.176106	-0.024199	H	1.860563	-2.175807	-0.04315
H	-0.607975	-2.077469	-0.049024	H	-0.607819	-2.077569	-0.05325

4-aminophenyl-carbodiimide **4p**

	4p-a				4p-s		
C	-0.593083	0.376011	-0.015395	C	-0.593096	0.375940	-0.016074
C	0.340419	1.415170	0.002487	C	0.340417	1.415129	0.001240
C	1.702491	1.145198	0.018351	C	1.702559	1.145299	0.010450
C	2.172885	-0.175983	0.012744	C	2.173072	-0.175866	0.001594
C	1.231188	-1.216740	-0.006013	C	1.231407	-1.216629	-0.020444
C	-0.130019	-0.945371	-0.020493	C	-0.129884	-0.945345	-0.028221
N	-1.960308	0.708485	-0.039735	N	-1.960420	0.708415	-0.029940
C	-2.962019	0.023235	0.016453	C	-2.962058	0.022949	0.024678
N	-4.026209	-0.578191	-0.088331	N	-4.027004	-0.577035	-0.080795
N	3.540266	-0.451042	0.084864	N	3.541548	-0.449501	-0.047482
H	-4.551700	-0.804554	0.748612	H	-4.548649	-0.811528	0.756283
H	-0.016055	2.438151	0.002147	H	-0.016166	2.438055	0.007216
H	1.570999	-2.247503	-0.006185	H	1.571406	-2.247181	-0.037993
H	-0.840180	-1.764968	-0.041640	H	-0.840027	-1.764987	-0.048277
H	3.824912	-1.357396	-0.255851	H	4.148676	0.280132	0.295275
H	4.153439	0.285588	-0.231355	H	3.820065	-1.363527	0.277174
H	2.411174	1.966802	0.037875	H	2.411327	1.967018	0.018506

5-(3-aminophenyl)-tetrazole **1m**

1m''-a

N	2.307832	0.968461	-0.010477
N	3.414437	0.238901	-0.003590
N	3.213388	-1.071267	0.009467
N	1.919953	-1.226601	0.011624
C	1.367964	0.022995	-0.000725
C	-0.075584	0.282021	-0.002136
C	-0.961896	-0.797768	-0.010723
C	-2.345894	-0.584863	-0.008661
C	-2.826148	0.733677	0.000421
C	-1.937881	1.804269	0.009413
C	-0.562430	1.595168	0.007882
N	-3.227638	-1.664356	-0.074109
H	-2.860399	-2.553024	0.232177
H	-4.161672	-1.493705	0.267310
H	4.339946	0.644230	-0.008989
H	-0.566460	-1.806834	-0.022607
H	-3.896163	0.915078	-0.004702
H	-2.326935	2.816328	0.017897
H	0.127097	2.428974	0.015681

1m'-a

N	2.363753	0.851214	-0.195794
N	3.550040	0.207852	-0.125922
N	3.269390	-1.024833	0.121290
N	1.930023	-1.215162	0.215987
C	1.364573	-0.035544	0.016474
C	-0.069229	0.259237	0.019726
C	-0.965868	-0.809806	-0.044414
C	-2.347759	-0.584095	-0.042193
C	-2.815333	0.739476	0.023147
C	-1.919360	1.798956	0.091711
C	-0.545184	1.575025	0.093152
N	-3.236270	-1.646562	-0.161669
H	-2.883923	-2.558488	0.087113
H	-4.179057	-1.485583	0.158350
H	2.335277	1.835574	-0.412170
H	-0.574503	-1.818767	-0.099687
H	-3.883138	0.931952	0.018004
H	-2.298322	2.812741	0.151889
H	0.134070	2.415513	0.173643

1m''-s

N	1.925388	-1.204678	-0.001724
N	3.215640	-0.899828	0.000601
N	3.476158	0.400056	0.003534
N	2.315213	0.990349	0.003384
C	1.367619	0.006402	-0.000117
C	-0.076715	0.260600	-0.001845
C	-0.973497	-0.811568	-0.007425
C	-2.354560	-0.586378	-0.006591
C	-2.822888	0.737163	-0.002496
C	-1.925543	1.799129	0.003512
C	-0.551288	1.577558	0.003789
N	-3.248417	-1.657247	-0.069077
H	-2.893006	-2.546400	0.249602
H	-4.180766	-1.472384	0.269859
H	3.945951	-1.597990	-0.001037
H	-0.590956	-1.825974	-0.016626
H	-3.891348	0.927617	-0.008926
H	-2.305017	2.814781	0.008188
H	0.148493	2.402345	0.008947

1m'-s

N	2.017874	-1.095461	-0.281071
N	3.351049	-0.910260	-0.159513
N	3.505174	0.315848	0.204179
N	2.311434	0.948787	0.323490
C	1.380309	0.060328	0.017347
C	-0.066771	0.280149	-0.004073
C	-0.957018	-0.796662	0.070708
C	-2.341440	-0.585609	0.041496
C	-2.816647	0.730751	-0.065371
C	-1.927423	1.797412	-0.130727
C	-0.552079	1.589334	-0.099877
N	-3.225934	-1.662628	0.066300
H	-2.878109	-2.517789	0.473678
H	-4.168938	-1.453249	0.358001
H	1.658761	-1.979566	-0.606688
H	-0.586357	-1.811564	0.177429
H	-3.886093	0.912028	-0.094744
H	-2.314723	2.806923	-0.207907
H	0.144692	2.414997	-0.150489

C-(3-aminophenyl)-nitrilimine **2m**

2mA-aa

C	-0.414317	-0.064634	-0.064799
C	0.680369	-0.936663	-0.039839
C	1.989320	-0.442730	0.023059
C	2.182372	0.947338	0.042755
C	1.093165	1.812898	0.004641
C	-0.208202	1.326297	-0.043341
C	-1.748005	-0.606631	-0.134416
N	-2.896520	-0.361975	0.026100
N	-4.131351	-0.157340	-0.024308
N	3.073125	-1.314608	0.114765
H	-4.550001	-0.402605	0.876818
H	0.501883	-2.005636	-0.067373
H	2.916377	-2.254874	-0.216139
H	3.965858	-0.945246	-0.176119
H	3.190185	1.346976	0.089906
H	1.265603	2.883309	0.017535
H	-1.054896	2.000283	-0.068886

2mA-as

C	-0.414314	-0.066529	-0.066582
C	0.681693	-0.936999	-0.053296
C	1.990205	-0.441571	0.010123
C	2.181164	0.948656	0.037475
C	1.090441	1.812824	0.011728
C	-0.210300	1.324541	-0.034772
C	-1.747779	-0.610426	-0.130900
N	-2.895783	-0.362527	0.029498
N	-4.130425	-0.157046	-0.021497
N	3.079565	-1.310369	-0.021510
H	-4.548076	-0.390851	0.883152
H	0.505110	-2.005671	-0.098206
H	2.901890	-2.259316	0.271343
H	3.948851	-0.947872	0.339973
H	3.188945	1.349678	0.071982
H	1.261333	2.883342	0.033382
H	-1.058215	1.997316	-0.049724

2mA-sa

C	-0.404648	0.512433	-0.067253
C	0.285000	-0.710297	-0.049988
C	1.682590	-0.740000	0.006989
C	2.380575	0.478433	0.065259
C	1.694254	1.687223	0.052348
C	0.305018	1.720838	-0.024296
C	-1.842609	0.524025	-0.150401
N	-2.830100	-0.106745	0.022449
N	-3.909099	-0.743754	-0.015991
N	2.365587	-1.953513	0.071622
H	-4.403248	-0.620982	0.871684
H	-0.275124	-1.638497	-0.081070
H	1.874177	-2.760132	-0.283419
H	3.333687	-1.933571	-0.211816
H	3.464286	0.470616	0.121631
H	2.253058	2.615274	0.093130
H	-0.232632	2.659459	-0.052645

2mA-ss

C	-0.405280	0.497324	-0.084757
C	0.298171	-0.717764	-0.076131
C	1.695426	-0.731807	-0.005649
C	2.379063	0.493535	0.074323
C	1.678458	1.694143	0.072550
C	0.289971	1.713400	-0.019866
C	-1.840918	0.491291	-0.179775
N	-2.837525	-0.113414	0.023335
N	-3.922182	-0.743362	0.018559
N	2.398330	-1.933706	-0.064246
H	-4.432459	-0.538428	0.881418
H	-0.250446	-1.651544	-0.133280
H	1.882471	-2.765313	0.181296
H	3.329537	-1.924423	0.323857
H	3.462868	0.497777	0.128327
H	2.225731	2.628018	0.132627
H	-0.257408	2.646551	-0.041962

C-(3-aminophenyl)-nitrilimine **2m**

2mP-a

C	0.406296	0.249533	-0.006921
C	-0.490705	-0.832199	-0.011616
C	-1.871821	-0.609904	-0.005946
C	-2.349304	0.711221	0.002553
C	-1.456220	1.777238	0.007110
C	-0.081895	1.570141	0.002347
C	1.799842	0.001134	-0.010924
N	2.948266	-0.208762	0.001437
N	4.145836	-0.617045	0.010306
N	-2.758347	-1.683229	-0.066235
H	4.767070	0.193381	0.020735
H	-0.105993	-1.845548	-0.024820
H	-2.401753	-2.575440	0.241608
H	-3.694830	-1.503912	0.263312
H	-3.418188	0.895886	-0.000186
H	-1.840464	2.791081	0.014834
H	0.606713	2.404814	0.006339

2mP-s

C	0.406855	0.207672	0.004880
C	-0.518790	-0.849477	-0.001682
C	-1.893405	-0.590705	-0.007437
C	-2.335514	0.742474	-0.006604
C	-1.414338	1.784381	0.001482
C	-0.046009	1.541124	0.006836
C	1.795930	-0.064550	0.011422
N	2.944567	-0.273738	0.000020
N	4.209099	-0.315368	-0.001099
N	-2.808389	-1.640653	-0.071071
H	4.503983	-1.292308	-0.039198
H	-0.162418	-1.873338	-0.007798
H	-2.479715	-2.538764	0.250515
H	-3.742230	-1.432726	0.249336
H	-3.399074	0.955476	-0.017398
H	-1.771263	2.808169	0.003429
H	0.665398	2.356284	0.012778

3-aminophenyl-1*H*-diazirine **3m**

	**3m-a**				**3m-s**		
C	0.566973	0.099795	-0.010224	C	0.566993	0.099746	-0.015018
C	-0.442887	-0.868843	0.007718	C	-0.442951	-0.868970	-0.007286
C	-1.786761	-0.483562	0.014026	C	-1.786876	-0.483673	0.002257
C	-2.090586	0.890819	0.004569	C	-2.090589	0.890837	0.001556
C	-1.080913	1.845138	-0.015369	C	-1.080806	1.845174	-0.007239
C	0.258698	1.463860	-0.022137	C	0.258799	1.463888	-0.015604
C	1.953389	-0.311781	-0.017014	C	1.953437	-0.311784	-0.016828
N	2.717950	-1.296586	-0.047906	N	2.718216	-1.296602	-0.041406
N	3.236533	0.280048	-0.065831	N	3.236900	0.279876	-0.059973
N	-2.802075	-1.432403	0.087026	N	-2.802958	-1.432675	-0.045952
H	3.536684	0.458689	0.898664	H	3.532072	0.460696	0.905688
H	-0.174138	-1.919754	0.019688	H	-0.174300	-1.919956	-0.012998
H	-2.564678	-2.367756	-0.207164	H	-3.710294	-1.138691	0.282033
H	-3.713079	-1.140451	-0.232899	H	-2.561754	-2.367204	0.247245
H	-3.129433	1.204728	0.015754	H	-3.129498	1.204717	0.001468
H	-1.343778	2.896584	-0.027486	H	-1.343613	2.896704	-0.009901
H	1.054089	2.197986	-0.048987	H	1.054242	2.198238	-0.033235

3-aminophenyl-carbodiimide **4m**

	**4m-a**				**4m-s**		
C	0.448354	-0.162889	-0.022887	C	0.448353	-0.163005	-0.023376
C	-0.697497	-0.956722	-0.003166	C	-0.697741	-0.956724	-0.016923
C	-1.969670	-0.370637	0.016556	C	-1.969940	-0.370551	0.002227
C	-2.070421	1.028858	0.016679	C	-2.070433	1.028979	0.010503
C	-0.920116	1.811133	-0.005941	C	-0.919869	1.811127	0.001158
C	0.345749	1.234024	-0.025501	C	0.345988	1.233920	-0.015370
N	1.689568	-0.828917	-0.051352	N	1.689571	-0.829215	-0.045709
C	2.825720	-0.399713	0.016155	C	2.825736	-0.399630	0.019487
N	4.000869	-0.069751	-0.074171	N	4.001038	-0.070807	-0.072760
N	-3.112322	-1.167926	0.092774	N	-3.114213	-1.167202	-0.046395
H	4.569724	0.015551	0.759749	H	4.569310	0.022729	0.760633
H	-0.584689	-2.034828	-0.000200	H	-0.585244	-2.034747	-0.032948
H	-3.011130	-2.117109	-0.234540	H	-3.963119	-0.741440	0.294281
H	-3.969293	-0.740105	-0.224605	H	-3.004927	-2.119153	0.269760
H	-3.048217	1.498359	0.037630	H	-3.048374	1.498588	0.017581
H	-1.013602	2.891500	-0.010040	H	-1.013141	2.891509	0.006219
H	1.237682	1.848467	-0.050111	H	1.238158	1.848386	-0.027708
